# Two patterns in apical dendrite extensions of projection neurons within cerebral cortex of reeler mutant mice

**DOI:** 10.3389/fnana.2025.1560972

**Published:** 2025-05-30

**Authors:** Ryoichi Ichikawa

**Affiliations:** ^1^Department of Anatomy, Sapporo Medical University School of Medicine, Sapporo, Japan; ^2^Department of Molecular Anatomy, Hokkaido University School of Medicine, Sapporo, Japan

**Keywords:** cerebral cortex, reeler mutant mice, corticospinal neurons, corticothalamic neurons, corticocallosal projection neurons, apical dendrite, thalamocortical fiber, corticocallosal fiber 9

## Abstract

**Introduction:**

Pyramidal-like projection neurons in the cerebral cortex exhibit layer-specific positioning of their cell bodies and target specific cortical regions with their apical dendrites. Reeler mutant mice, which lack the gene for the reelin protein gene secreted by Cajal–Retzius cells and have their projection neurons scattered throughout the cortex, display relatively intact global and local neuronal network connections compared with wild-type mice. The irregular morphologies of these cells, which extend their apical dendrites in a neuron-disoriented direction, are thought to compensate for the malposition of the neurons. I aimed to investigate the projection target-specific regulation of this apical dendrite extension pattern in reeler mice.

**Methods:**

To this end, three types of projection neurons–corticospinal (CS), corticothalamic (CT), and corticocallosal (CC) neurons–were evaluated using retrograde labeling techniques.

**Results and discussion:**

Reeler CS neurons displayed a congregation pattern of apical dendritic terminal tips in a specific upper cortical zone, whereas reeler CC neurons exhibited a dispersed pattern of scattered tips throughout the cortex. However, reeler CT neurons showed a hybrid pattern, exhibiting characteristics of both congregation- and dispersion-type neurons. Moreover, apical dendrite extension of these projection neurons follows either a congregation or dispersion mode from postnatal day 0 (P0), which subsequently defines their terminal tip positioning by P8. Thus, this early patterning of apical dendrite arborization in reeler projection neurons likely contributes to the formation of projection target-specific neuronal connections during the first two postnatal weeks.

## 1 Introduction

Projection neurons in the cerebral cortices exhibit a characteristic pyramidal-like shape due to the radial extension of the apical dendrites (ADs) and horizontal extension of basal dendrites. Moreover, these neurons are located at their cell body centers (CBCs) within projection target-specific layers. The final positions of the apical dendrite terminal tips (ADTTs) depend on the projection target as well as the CBC position layer. For instance, corticocallosal projection neurons (CC neurons) are located in layers 2 + 3 and 5, wherein those in layer 2 + 3 extend beneath the pia matter, whereas those at layer 5 settle within layer 2 + 3 or 4 or extend beneath the pia matter with poor arbor (Harris and Shepherd, 2015). During the early developmental stage in wild-type mice, migrating neuroblasts destined to become corticospinal (CS), corticothalamic (CT), and CC neurons are guided by the radial glial cells towards the marginal zone. Their leading processes come in contact with the Cajal–Retzius cells, and subsequent neuroblasts stop migrating below them, ultimately positioning their ADTTs beneath the pia mater (Katsuyama and Terashima, 2009; Marin-Padilla, 2015). Thereafter, some neurons maintain their ADTTs in these regions, whereas others retract the distal portion of their ADs and relocate their ADTTs within the cerebral cortices during the first 2 postnatal weeks in the manner depending upon the projection target.

Reeler mutant mice lack the gene expressing reelin, a protein secreted by Cajal–Retzius cells crucial for neuron migration (Caviness and Sidman, 1972; D’Arcangelo et al., 1995; Hirotsune et al., 1995; Ogawa et al., 1995; Tissir and Goffinet, 2003; Guy and Staiger, 2017). Morphological studies have revealed that neurons destined to form specific layers are scattered throughout the cortex (Terashima et al., 1983; Terashima et al., 1985; Dekimoto et al., 2010; Guy and Staiger, 2017). Notably, although reeler mice exhibit severe ataxia, it is generally accepted that their global and local neuronal connections are relatively intact (Lemmon and Pearlman, 1981; Simmons and Pearlman, 1982; Silva et al., 1991; Guy et al., 2016), which is further supported by morphological studies on the barrel structure (Welt and Steindler, 1977; Wagener et al., 2010; Wagener et al., 2016; Guy et al., 2015) and granular retrosplenial cortices (Ichinohe et al., 2008; Guy and Staiger, 2017). However, most projection neurons display irregular pyramidal shapes. In contrast to wild-type projection neuron ADs, which exhibit a homogenous radial orientation toward the pia matter, reeler projection neuron ADs demonstrate a heterogenous orientation following a disoriented direction (Landrieu and Goffinet, 1981; Terashima et al., 1983; Terashima et al., 1985). It is possible that the abnormal AD extension observed in reeler projection neurons is contributed by the formation of appropriate synaptic connections with afferent fiber inputs depending upon the projection target.

To evaluate projection target-specific patterns of AD extensions in the cerebral cortices of reeler mutant mice, CS, CT, and CC neurons were examined using retrograde labeling techniques (biocytin and postmortem DiI). This allowed the assessment of the intracortical position and morphology of the retrograde-labeled neurons, particularly the radial positions of the ADTTs and final branching point of AD main shaft (ADFBP), radial distance between the CBC and ADTT, and path length from CBC to the ADTT. These parameters were then correlated with the CBC positions. Furthermore, developmental changes in the correlations between the three types of reeler projection neurons were evaluated.

## 2 Materials and methods

### 2.1 Animals

All experiments adhered to the guidelines and approval of the Committee on Animal Care and Welfare, Hokkaido University School of Medicine, Committee for Animal Research, and the Animal Care and Welfare Committee of Sapporo Medical University. Heterozygous reeler mutant mice [reelin (+/−)] were obtained from the Jackson Laboratory (Bar Harbor, ME) and bred in our facility (Hokkaido University School of Medicine). Littermates from these heterozygotes were used at postnatal day 0 (P0) or raised until P2, P4, P8, and 6 weeks. Genotyping (homozygous or not) of littermates was performed using polymerase chain reaction at each time point (D’Arcangelo et al., 1995; Yamamoto et al., 2003). Three pairs of age-matched reeler and wild-type (reelin +/+) mice of either sex, from the same litter, were used to label each target-specific cortical neuron at each timepoint.

**Figure 1:**
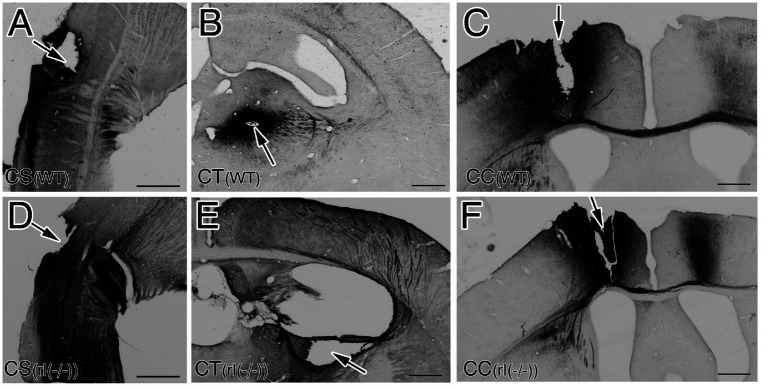
Injection sites of biocytin in wild-type mice (WT) **(A–C)** and reeler mice [rl(−/−)] **(D–F)**. **(A,D)** Sagittal sections around the pyramidal decussation at the transition between the medulla oblongata and the spinal cord to label corticospinal neurons (CS). **(B,E)** Coronal secstions including the ventral anterior and ventral lateral (VA + VL) nucleus of the right thalamus to label corticothalamic neurons (CT). **(C,F)** Coronal sections including the left primary motor cortex (M1) to label corticocallosal neurons (CC). Arrows indicate injection directions or sites. Note. Biocytin is injected throughout all layers of left M1. Scale bar: 500 μm.

**Figure 2:**
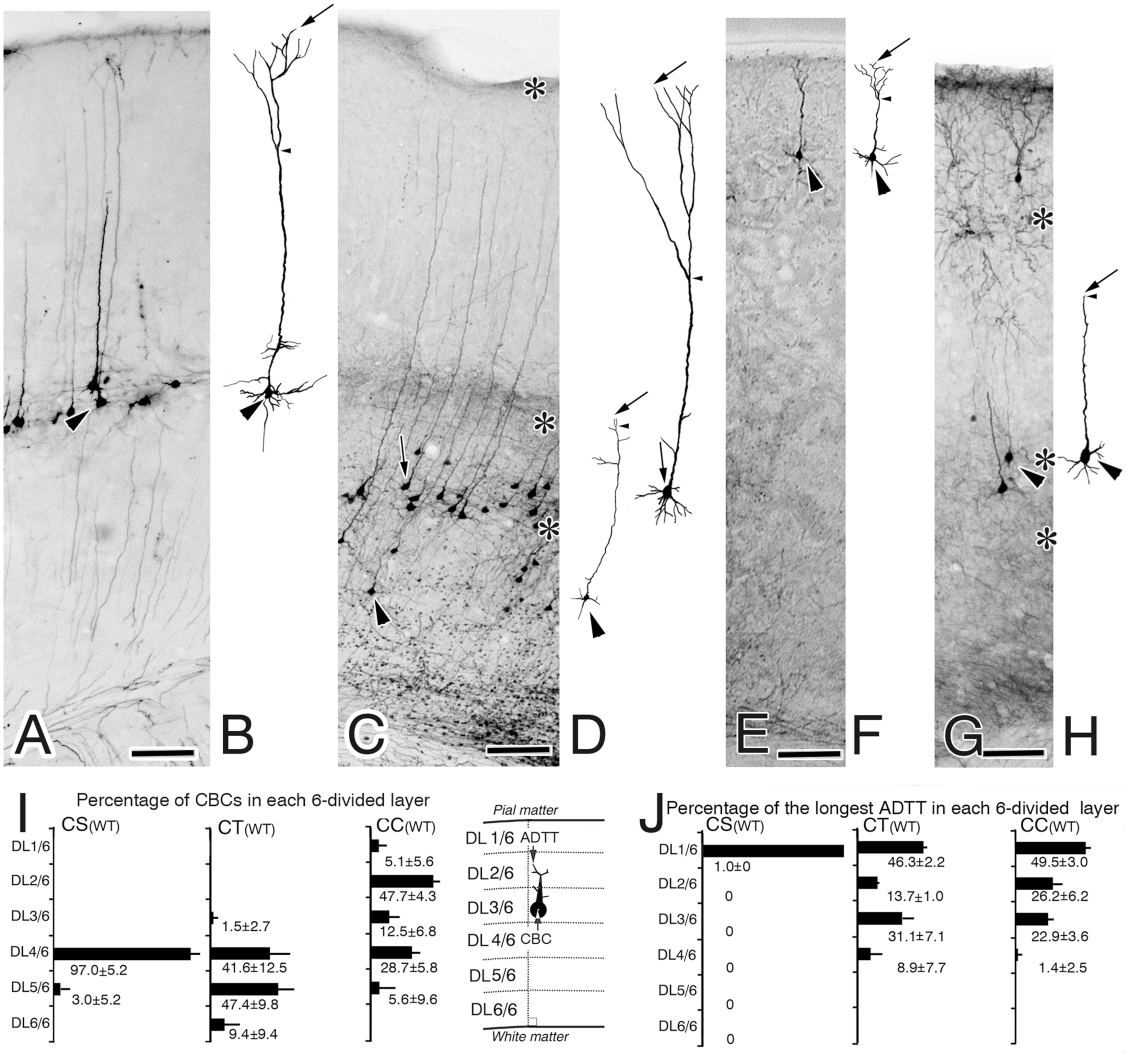
Biocytin-retrograde-labeled neurons in the right primary motor cortex of wild-type (WT) mice **(A,C,E,G)** and corresponding reconstructed images of these labeled neurons **(B,D,F,H)**. **(A,B)** Corticospinal neurons (CS). **(C,D)** Corticothalamic neurons (CT). **(E,H)** Corticocallosal neurons (CC). The images in **B,D,F,H** show reconstructions of the neuron indicated by the large arrowhead in **A,C,E,G**, recpectively. In **B,D,F,H** arrows indicate the tips of the longest apical dendrite (ADTTs), small arrowheads indicate the final branching point of main shaft of apical dendrites. **(I)** Percentages of CS, CT, and CC, with CBCs located in each of the divided six cortical layers (from the pia mater, DL1/6, DL2/6, DL3/6, DL4/6, DL5/6, DL6/6), to the total amount of CS, CT, and CC. **(J)** Percentages of CS, CT, and CC, with ADTTs with the longest path length located in each of the divided six cortical layers. The labeled number is the average ± SD% from the three mice for each projection neuron. Schematic illustrations of measured parameters are on the drawn right sides of **(I)**. Scale bar: 100 μm.

CS, CT, and CC neurons in the right primary motor cortex (M1) of the two mice were labeled as follows. Each mouse was anesthetized using sevoflurane inhalation and secured in a stereotactic apparatus (SRS-6, Narishige, Tokyo, Japan). A glass pipette filled with 1 μm of 5% biocytin (Sigma-Aldrich, St. Louis, MO) in 0.05 M Tris (pH 7.6) buffer solution was injected using a micromanipulator (SM11, Narishige) into three sites: (1) the pyramidal decussation of the left cervical spinal cord from dorsal side to label CS neurons (Figures 1A,D), (2) the ventral anterior and ventral lateral (VA + VL) nucleus of the right thalamus to label CT neurons (Figures 1B,E; Supplementary Figures 1A–D), and (3) the left primary motor cortex to label CC neurons (Figures 1C,F; Supplementary Figures 1E–H). Following a 24-h survival period, each mouse was transcardially perfused with a mixed solution of 4.0% paraformaldehyde and 0.2% picrinic acid in 0.1 M phosphate buffered solution (PB, pH 7.4) under deep anesthesia by sevoflurane inhalation. The brain of each mouse was subsequently removed and immersed in 30% sucrose in 0.1 M PB at 4°C for 48 h. To visualize the labeled neurons, coronal sections (50-μm thick) of the right cortex were prepared using a freezing microtome. These sections were then incubated overnight in streptavidin-horseradish peroxidase (Amersham Pharmacia Biotech, Buckinghamshire, UK) diluted with 0.1 M PB containing 1% Tween 20 and visualized using diaminobenzidine (DAB) and cobalt (Horikawa and Armstrong, 1988). All sections were placed on gelatin-coated glass slides, and some sections were stained with Nissl to identify the six cortical layers of wild-type brains and an inverted layer structure of reeler brains. These sections were mounted after dehydration with alcohol. Images were obtained using a Normarski interference contrast microscope (AX-80; Olympus, Tokyo, Japan).

CS, CT and CC neurons at postnatal day 0 (P0), P2, P4, and P8 were labeled using the postmortem 1,1′-dioctadecyl-3,3,3′,3′-tetramethylindocarbocyanine perchlorate (DiI) technique (Koester and O’Leary, 1992). Under anesthesia induced by sevoflurane inhalation, mice were transcardially perfused with a 4.0% paraformaldehyde in 0.1 M PB, and brains were then removed. For labeling, crystals of DiI (Sigma-Aldrich) were placed into the corresponding brain regions of pups, similar to the procedure conducted in 2-month-old mice. Following labeling, brains were incubated in the same fixed solution at 37°C for 2 months. To visualize labeled neurons, coronal sections (150-μm thick) of the right cortex were prepared using a microslicer (DKT-1500; Dosaka EM, Tokyo, Japan). Images were obtained using a confocal laser microscope (MRC-500; Bio-Rad, Hercules, CA).

### 2.2 Morphometrical procedure

The intracortical positions of CBCs, longest ADTTs with the longest path length in certain directions (e.g., total amount of dendritic cylinder lengths from CBC to the tip), the second ADTTs with the second longest path length, the third ADTTs with the third longest path length, ADFBP, and ADTT path lengths (Supplementary Figure 2) of labeled CS, CT, and CC neurons were all measured in 6-month-old wild-type and reeler mutant mice using personal computers connected with a digitizer analysis machine, where images were drawn using light microscopy and camera lucida equipment (BH-10, Olympus). Similar measurements were performed on postmortem DiI-labeled neurons from P0, P2, P4, and P8-old mice using the equipped software (MRC-500, Bio-Rad).

For the 6-month-old mice, all the biocytin- or DiI-labeled neurons whose cell bodies were located in a single section of the right M1 were counted. The outline of the right M1 was initially drawn at 100 × magnification (low-magnification drawing), marking the CBC, the longest ADTT, the second ADTT, the third ADTT and the ADFBP positions of the selected neurons. If the ADTTs were in a section adjacent to the CBC, its positions were marked in the CBC-containing section based on tracing from adjacent sections. If the three types of ADTTs and ADFBP of the neuron could not be traced from the CBC, the neuron was excluded from ADTT-related measurements. The numbers of the neurons measured for the three types of ADTT and ADFBP were shown in Supplementary Table 1 and 2. Dendritic figures from the neurons were then precisely traced at 1000 × magnification (high-magnification drawing). If the neuron spanned multiple adjacent sections, the entire figure of the CB and dendrites was reconstructed by combining features from adjacent sections. A custom software for implementing path length calculations (Capowski, 1989) was developed and run on a personal computer (ThinkPad 560E; IBM, Armonk, NY) connected to a digitizer analysis machine (KW4620-RS; Graphtech, Tokyo, Japan). Utilizing this machine, the following parameters were measured: the intracortical heights (radial distance from the cortical base) of CBCs, the three types of ADTTs and ADFBP, widths of the CBC- or ADTT-containing regions, the path length of longest ADTT and the angle between the radial axis and the vector from the CBC to the three types of ADTT.

For P0-, P2-, P4-, and P8-old mice, these parameters were measured using the same software at low or high magnification (MRC-500, Bio-Rad). One of these measurements included cortical width, which is defined as the distance from the gray matter–white matter boundary to the upper border of the gray matter, including the subplate.

**Figure 3:**
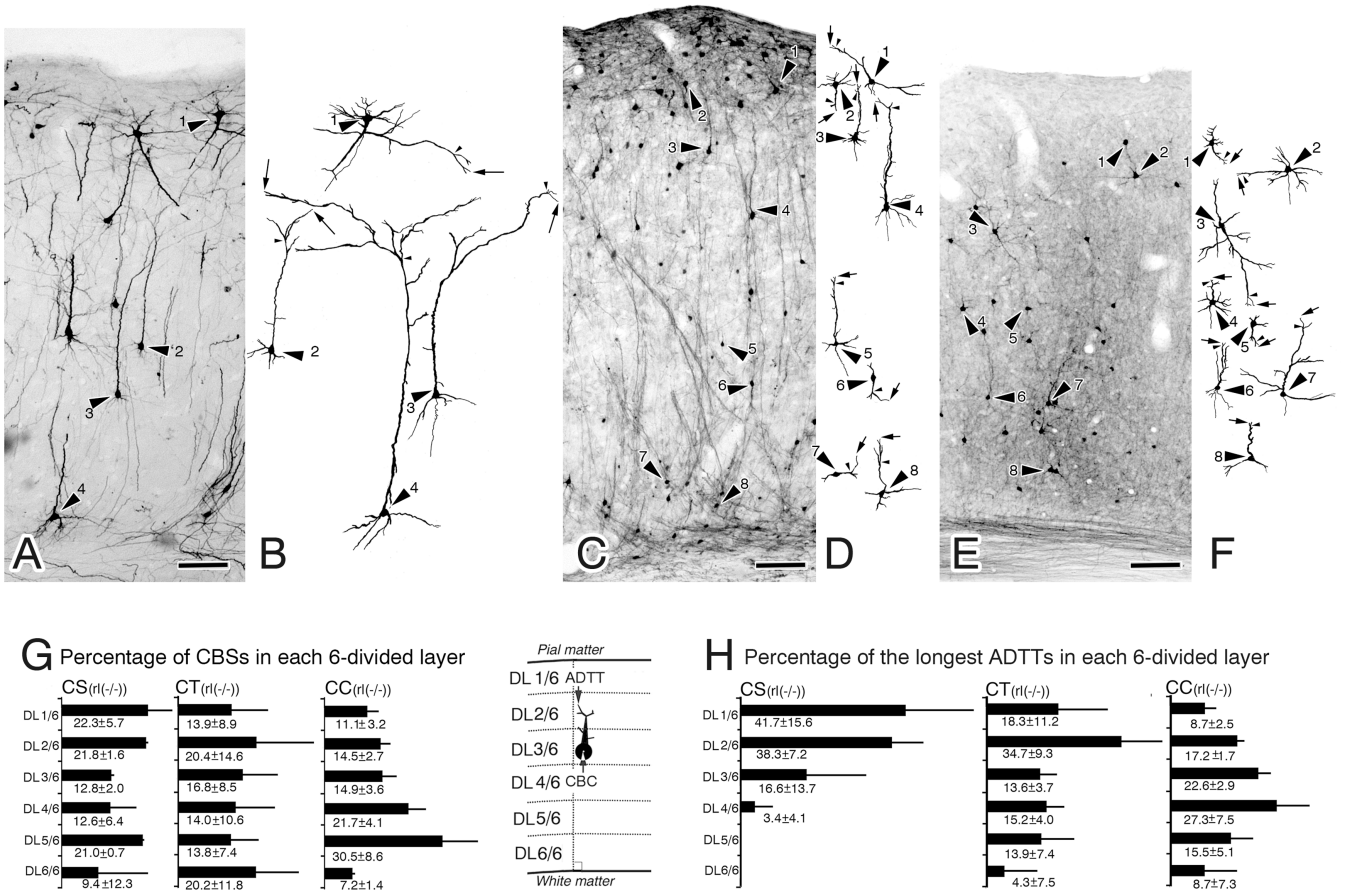
Biocytin-retrograde-labeled neurons in the right primary motor cortex of reeler mutant mice [rl(−/−)] **(A,C,E)** and reconstructed images from the labeled neurons **(B,D,F)**. **(A,B)** Corticospinal neurons (CS). **(C,D)** Corticothalamic neurons (CT). **(E,F)** Corticocallosal neurons (CC). The images in **B,D,F** show reconstructions of the neuron indicated by the large arrowhead in **A,C,E**, recpectively. In **B,D,F**, arrows indicate the tips of the longest apical dendrite (ADTTs), small arrowheads indicate the final branching point of main shaft of apical dendrites. **(G)** Percentages of CS, CT, and CC, with CBCs located in each of the divided six cortical layers (from the pia mater, DL1/6, DL2/6, DL3/6, DL4/6, DL5/6, DL6/6), to the total amount of CS, CT, and CC. **(H)** Percentages of CS, CT, and CC, with ADTTs with the longest path length located in each of the divided six cortical layers. The labeled number is the average ± SD% from the three mice for each projection neuron. Schematic illustrations of measured parameters are on the drawn right sides of **(H)**. Scale bar: 100 μm.

Intracortical distributions of CBC and longest ADTT were visualized in bar graphs, wherein the M1 area was divided into six layers tentatively named from the pia mater as DL1/6, DL2/6, DL3/6, DL4/6, DL5/6, and DL6/6 (Figures 2I–J, 3G–H; Supplementary Figure 2). The number of CBCs and longest ADTTs located within these layers were counted in each mouse, and their percentages to the total number were calculated. The percentages from three mice were averaged. Afterwards, the intracortical positions of the CBCs, three types of ADTTs and ADFBP of individual neurons were quantified by dividing their height by the cortical width (Supplementary Figure 2). Radial distances of longest ADTTs were calculated by subtracting the CBC height from longest ADTT height (Supplementary Figure 2). From these calculations, six types of scatter diagrams correlating intracortical CBC positions with longest ADTT positions, longest ADTT radial distances, longest ADTT path lengths, ADFBP positions, the second ADTT positions and the third ADTT positions were generated. Pearson’s correlation coefficient test was used to assess these correlations in each mouse.

### 2.3 Statistical analysis

Statistical tests, including Pearson’s correlation and the generation of the scatter diagrams, were performed using a software package (BellCurve for Excel 2015, Social Survey Research Information, Tokyo, Japan). Correlations were considered significant if the three regression coefficients exceeded 0.50.

## 3 Results

### 3.1 Overview of CS, CT, and CC neurons in the sensorimotor cortices of adult wild-type and reeler mutant mice

Biocytin injections using a sharpened glass micropipette were utilized to visualize CS, CT, and CC neurons in the right M1, targeting the pyramidal decussation for CS neurons, the right VA + VL thalamic nucleus for CT neurons, and the left M1 for CC neurons. Brains with an intranuclear or intracortical injection site were selected for the visualization of retrogradely labeled projection neurons (Figure 1; Supplementary Figure 1). Notably, projection neurons within the M1 of right reeler mutant and wild-type mice exhibited good retrograde labeling. Outlines of the CBs and ADs of neurons were clearly traceable until reaching the ADTTs (Figures 2B,D,F,H, 3B,D,F). Alongside the retrogradely labeled CT and CC neurons, anterogradely labeled thalamocortical and callosal commissural fibers were also observed. Although wild-type thalamocortical fiber terminals were relatively restricted to layer 1, 4, and 6 (asterisks in Figure 2C), reeler thalamocortical fiber gathered in superficial zone and descended with terminating a broader distribution in the upper and middle cortical zones (Figure 3C). Similarly, wild-type callosal commissural fibers originating from the contralateral hemisphere terminated densely in layer 2 + 3, 5 and 6 (asterisks in Figure 2G), whereas reeler fibers terminated throughout the middle and lower cortical zones (Figure 3E).

Genotypic differences were observed upon comparing the CBC and longest ADTT heights of reeler CS, CT, and CC neurons with those of their wild-type counterparts. To quantify the intracortical distribution of CBCs and longest ADTTs, the M1 was divided into six layers (DL1/6-DL6/6), calculating the percentages of labeled neurons with CBCs or longest ADTTs located within each layer (Figures 2I–J, 3G–H). For CBCs, wild-type brains showed distributions restricted in the following layers: CS neurons in DL4/6–5/6; CT neurons in DL3/6–6/6, with the highest densities in DL4/6–5/6 (approximately 90%); and CC neurons in DL1/6–5/6, with the highest densities in DL2/6 (47.7 ± 4.3%, n = 3) and DL4/6 (28.7 ± 5.6%) (Figure 2I). Conversely, reeler CBCs of these neurons showed a scattered distribution throughout the right M1 cortex, demonstrating heterogeneity in a projection target-specific manner (Figure 3G). For longest ADTTs, wild-type brains showed distributions restricted in the following layers: CS neurons in DL1/6; CT neurons in DL1/6–4/6, with the highest densities in DL1/6 (46.3 ± 2.2%, n = 3) and DL3/6 (31.1 ± 7.1%); and CC neurons in DL1/6–3/6, with the highest densities in DL1/6 (49.5 ± 3.0%, n = 3), DL2/6 (26.2 ± 6.2%), and DL3/6 (22.9 ± 3.6%) (Figure 2J). However, for longest ADTTs in reeler brains, CS neurons were located in DL1/6–4/6, showing higher densities in the upper zone (DL1/6 [41.7 ± 15.6%, n = 3], DL2/6 [38.3 ± 7.2%], DL3/6 [16.6 ± 13.7%]). CT neurons were scattered entirely in M1, with the highest zone in DL2/6 (34.7 ± 9.3%, n = 3), and CC neurons showed high-density distributions in DL3/6 (22.6 ± 2.9%, n = 3) and DL4/6 (27.3 ± 7.5%) (Figure 3H).

### 3.2 Two AD extension patterns in reeler projection neurons

**Figure 4:**
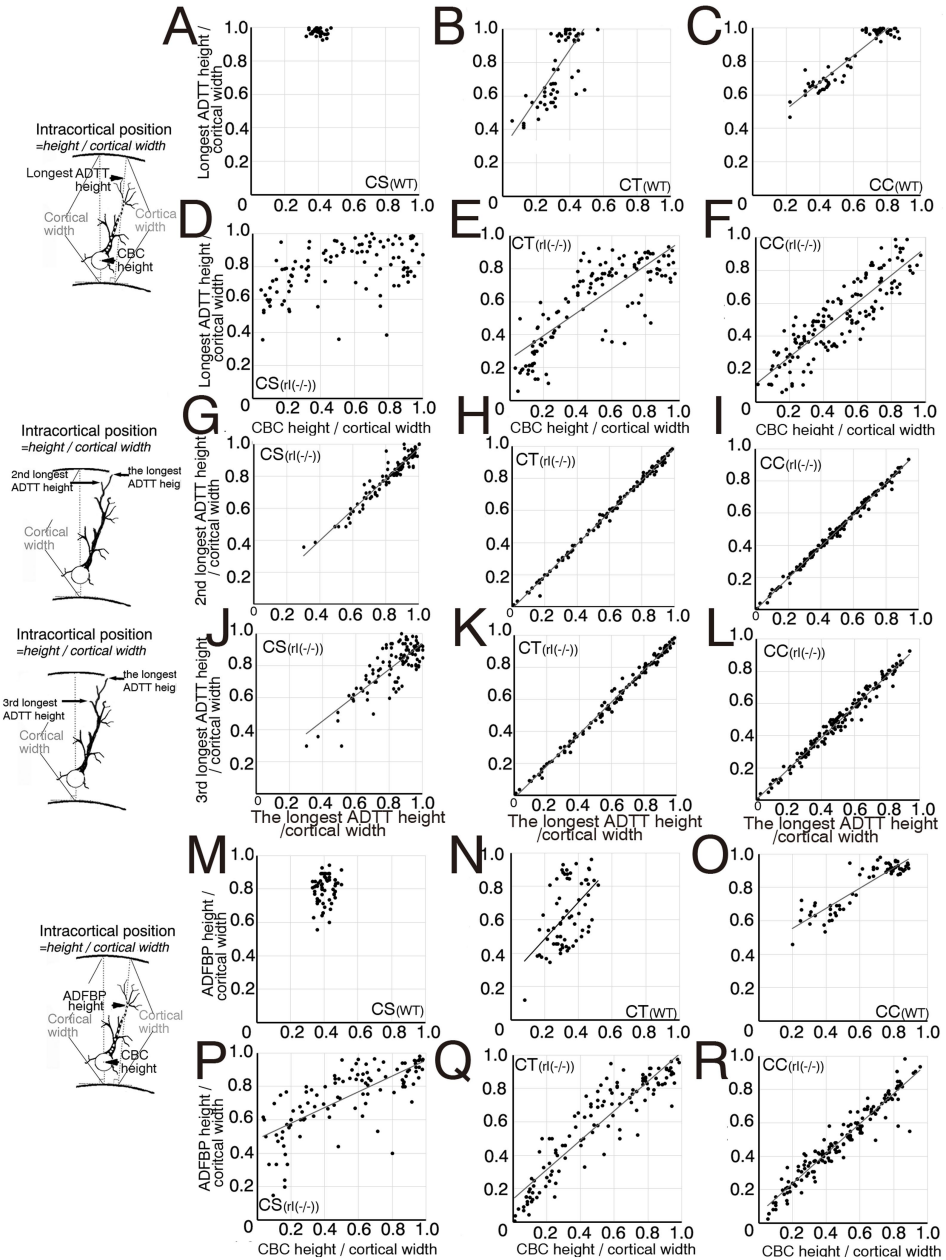
Scatter diagrams of three measured types for each projection neuron. **(A–F)** Between cell body center (CBC) height per cortical width and the longest apical dendrite terminal tip (ADTT) height with the longest path from CBC to ADTT height per cortical width in wild-type (WT) corticospinal neurons (CS) **(A)**, corticothalamic neurons (CT) **(B)**, and corticocallosal neurons (CC) **(C)**, and in reeler [rl(−/−)] CS **(D)**, CT **(E)**, and CC **(F)**. **(G–I)** Between the longest ADTT height per cortical width and the second longest ADTT height per cortical width in WT CS **(G)**, CT **(H)**, and CC **(I)**, and in rl(−/−) CS **(J)**, CT **(K)**, and CC **(L)**. **(J–L)** Between the longest ADTT height per cortical width and the third longest ADTT per cortical width in rl(−/−) CS (J), CT **(K)**, and CC **(L)**. **(M–R)** Between CBC height per cortical width and final branching point of main shaft of apical dendrite (ADFBP) height per cortical width in WT CS **(M)**, CT**(N)**, and CC **(O)**, and in rl(−/−) CS **(P)**, CT **(Q)**, and CC **(R)**. When the correlation coefficient exceeded 0.5 in three mice, regression lines were drawn using this formula. Schematic illustration of the measured parameters drawn on the left sides of between **A** and **D, G, J**, between **M** and **P**.

**Figure 5:**
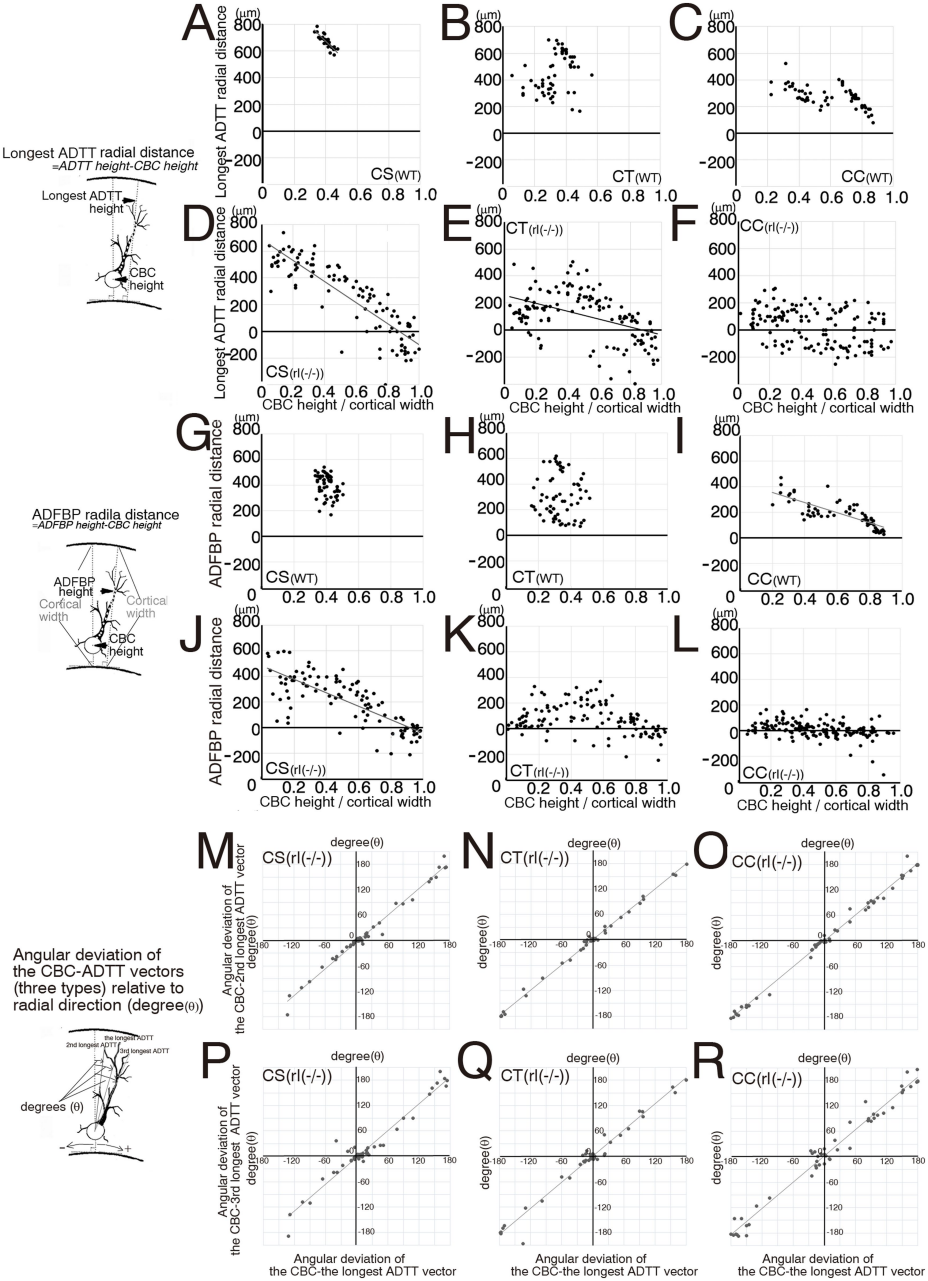
Scatter diagrams of three measured types for each projection. **(A–F)** Between cell body center (CBC) height per cortical width and the longest apical dendrite terminal tip (ADTT) radial distance per cortical width in wild-type (WT) corticospinal neurons (CS) **(A)**, corticothalamic neurons (CT) **(B)**, and corticocallosal neurons (CC) **(C)**, and in reeler [rl(−/−)] CS **(D)**, CT **(E)**, and CC **(F)**. **(G–L)** Between CBC height per cortical width and final branching point of main shaft of apical dendrite (ADFBP) radial distance per cortical width in WT CS **(G)**, CT **(H)**, and CC **(I)**, and in rl(−/−) CS **(J)**, CT **(K)**, and CC **(L)**. **(M–O)** Between angular deviation of the CBC-the longest ADTT vector to relative to radial direction and angular deviation of the CBC-the second longest ADTT vector to radial direction in rl(−/−) CS **(M)**, CT **(N)**, and CC **(O)**. **(P–R)** Between angular deviation of the CBC-the longest ADTT vector to relative to radial direction and angular deviation of the CBC-the third longest ADTT vector to radial direction in rl(−/−) CS **(P)**, CT **(Q)**, and CC **(R)**. When the correlation coefficient exceeded 0.5 in three mice, regression lines were drawn using this formula. Schematic illustration of the measured parameters drawn on the left sides of between **A** and **D**, between **G** and **J**, between **M** and **P**.

The correlation between the three types of intracortical ADTT positions, ADFBP positions, longest ADTT radial distances, ADFBP radial distances, and longest ADTT path length with intracortical CBC position was evaluated in wild-type and reeler CS, CT, and CC neurons (Figures 4, 5). The three types of ADTT positions of wild-type CS neurons were concentrated at the upper marginal zone in M1, regardless of the CBC position (Figures 4A, Supplementary Figures 3G,M). In contrast, ADFBP positions were more broadly distributed in upper cortical zone compared to the three types of ADTT positions (Figures 4M–O). These data suggested that longest ADTT radial distance and longest ADTT path length were positively correlated with CBC position (Figures 5A; Supplementary Figure 3A). Wild-type CT and CC neurons were divided into two groups: one group comprised neurons located in the upper zone with the three types of ADTTs terminating in the upper marginal zone, and the other comprised neurons located in the deeper zone with the three types of ADTTs terminating in DL2/6–5/6 (Figures 4B,C; Supplementary Figures 3H,I,N,O).

In the reeler cortex, the second longest, and third longest ADTTs originating from a single CS, CT, or CC neuron showed no significant difference in height compared to the longest ADTTs (Figures 4G–L). CS neurons demonstrated a dense clustering within a specific region of the upper cortical zone of M1, regardless of CBC position (Figure 4D, Supplementary Figures 3J,P). ADFBP positions in reeler CS neurons were positively correlated with CBC positions (Figure 4P), but a tendency for ADFBP positions was observed in a specific region of the upper cortical zone of M1 compared to CT and CC neurons (Figures 4Q,R). Furthermore, in reeler CS neurons, the radial distances of ADFBPs from CBC heights show a positive correlation with the position of CBC (Figure 5J). Notably, CS neurons with CBCs located in deeper cortical zone exhibit longer ADFBP distances compared to those in wild-type CS neurons (Figures 5G,J). These findings suggest that reeler CS neurons tend to maintain a long main shaft of the apical dendrite. These data suggest that the radial distances and path lengths of the three ADTT types are positively correlated with the position of CBC (Figures 5D; Supplementary Figure 3D). These data showed the tendency of the ADTTs in reeler CS neurons to congregate in a specific upper zone region, indicative of a congregation pattern. In contrast, the radial distances of the longest ADTT and ADFBP in reeler CC neurons were heterogeneous—within ranges of approximately 200 and 150 μm, respectively—regardless of CBC position, whereas those in wild-type CC neurons generally exceeded 300 and 200 μm, respectively (Figures 5C,F,I,L). These data indicated that a positive correlation between the three types of ADTT positions and CBC positions (Figure 4F; Supplementary Figures 3L,R) as well as a positive correlation between ADFBP positions and CBC positions (Figure 4R). These findings show that three types of ADTTs in reeler CC neurons exhibited a freely dispersive distribution within this range, indicative of a dispersion pattern. Finally, reeler CT neurons showed a positive correlation of the three types of ADTT and ADFBP positions to CBC positions (Figures 4E,Q; Supplementary Figures 3K,Q), and, though at milder than CS neurons, the positive correlation between longest ADTT radial distance and CBC positions (Figure 5E). These data suggested that a combination or intermediate of congregation and dispersion patterns. Specifically, the relationship of the longest ADTT and ADFBP radial distances to CBC positions of the neurons situated in the upper and middle cortical zones resembled that of reeler CS neurons, whereas the relationship of those in the deep zone resembled that of reeler CC neurons (Figures 5E,K). Thus, these findings support the combination of congregation- and dispersion-type neurons in reeler CT neurons.

The longest, second longest, and third longest ADTTs originating from a single CS, CT, or CC neuron were distributed within spatially confined intracortical layers of the reeler cortex (Figures 4G–L). To investigate the horizontal distribution pattern of these ADTTs, the radial and horizontal distances from the CBC to each ADTT were measured, and the angle of the vector from the CBC to each ADTT relative to the radial axis was calculated (Supplementary Figures 4A–I). A comparison of these angles among the three types of ADTTs revealed no significant differences (Figures 5M–R).

### 3.3 Two mode of developmental changes in the AD extensions of reeler projection neurons

**Figure 6:**
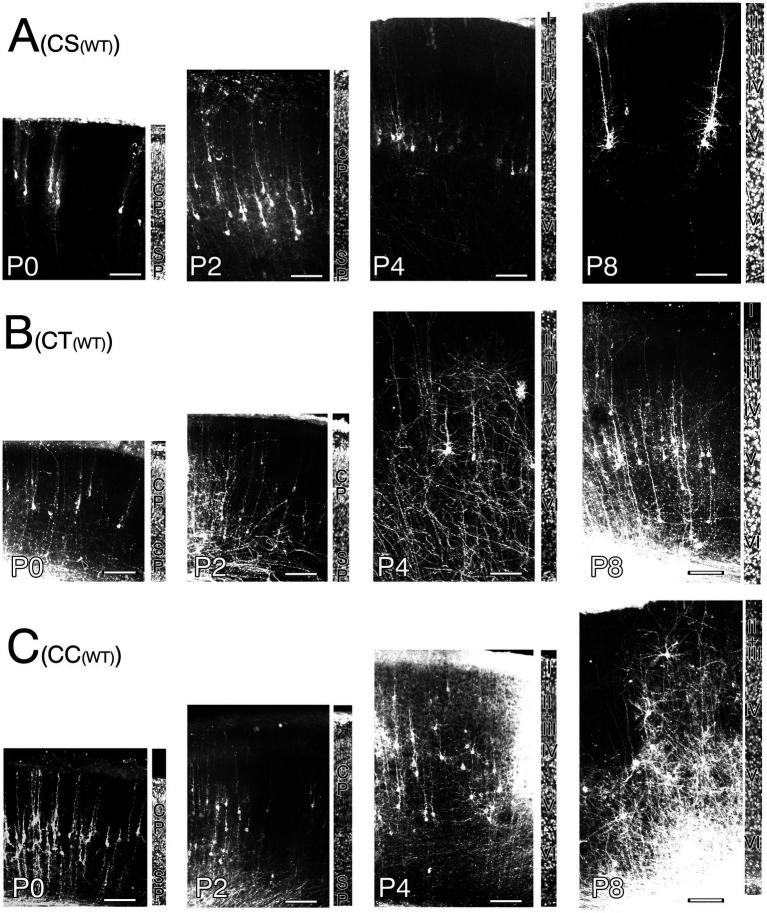
Postmortem DiI-retrograde-labeled neurons in the right primary motor cortex of wild-type mice (WT) appended with adjacent field of the same section counterstained to show cortical lamination from P0 to P8. **(A)** Corticospinal neurons (CS). **(B)** Corticothalamic neurons (CT). Note. malformed labeled neuron whose ADTT terminates at deep cortical zone is indicated by arrowheads. **(C)** Corticocallosal neurons (CC). Cortical plate (CP). Subplate (SP). layer 1(I). layer 2 (II). layer 3 (III). layer 4 (IV). layer 5 (V). layer 6 (VI). Scale bar: 100 μm.

**Figure 7:**
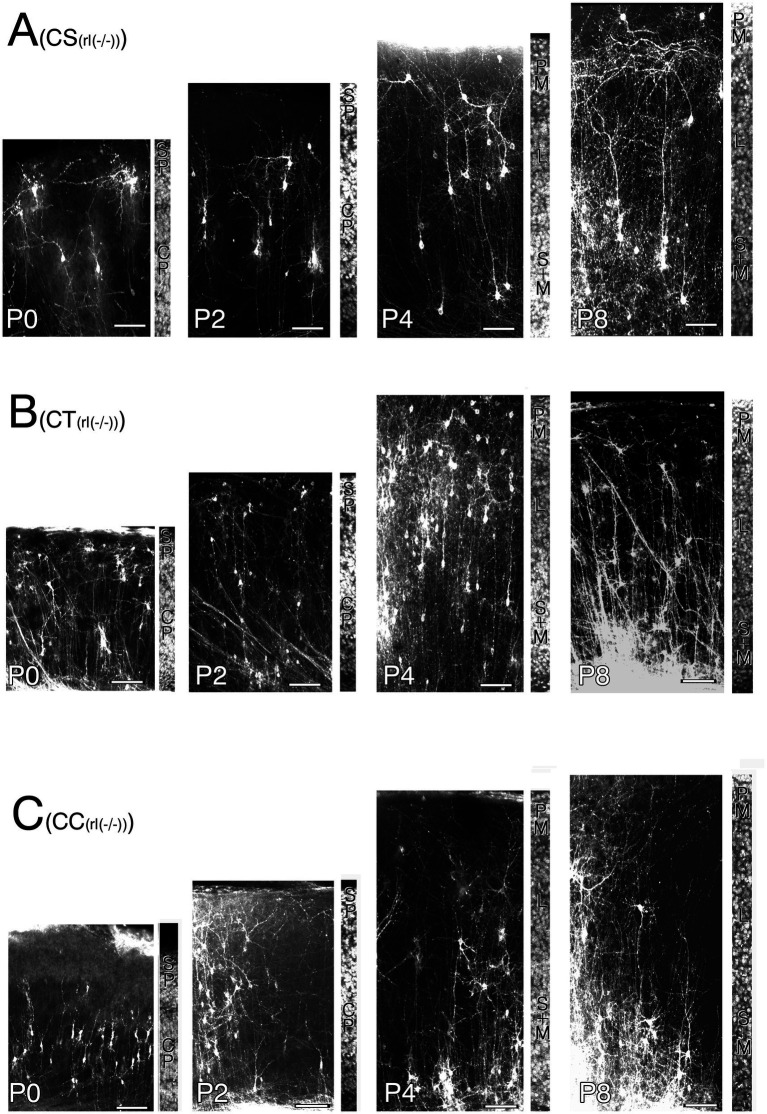
Postmortem DiI-retrograde labeled neurons in the right primary motor cortex of reeler mice [rl(−/−)] appended with adjacent field of the same section counterstained to show cortical lamination from P0 to P8. **(A)** Corticospinal neurons (CS). **(B)** Corticothalamic neurons (CT). **(C)** Corticocallosal neurons (CC). Cortical plate (CP). Subplate (SP). Polymorphic zone (PM). Large pyramidal cell zone (L). Small to Medium pyramidal cell zone (S + M). Scale bar: 100 μm.

To visualize the CS, CT, and CC neurons in the right M1 at P0, P2, P4, and P8, postmortem DiI crystals were inserted into the pyramidal decussation of paraformaldehyde-fixed removed brains, right VA + VL nucleus of the right thalamus, and left M1, respectively. Following a 2-month incubation period at 37°C, the labeled neurons were visualized using laser confocal microscopy (Figures 6, 7; Supplementary Figures 5–8).

**Figure 8:**
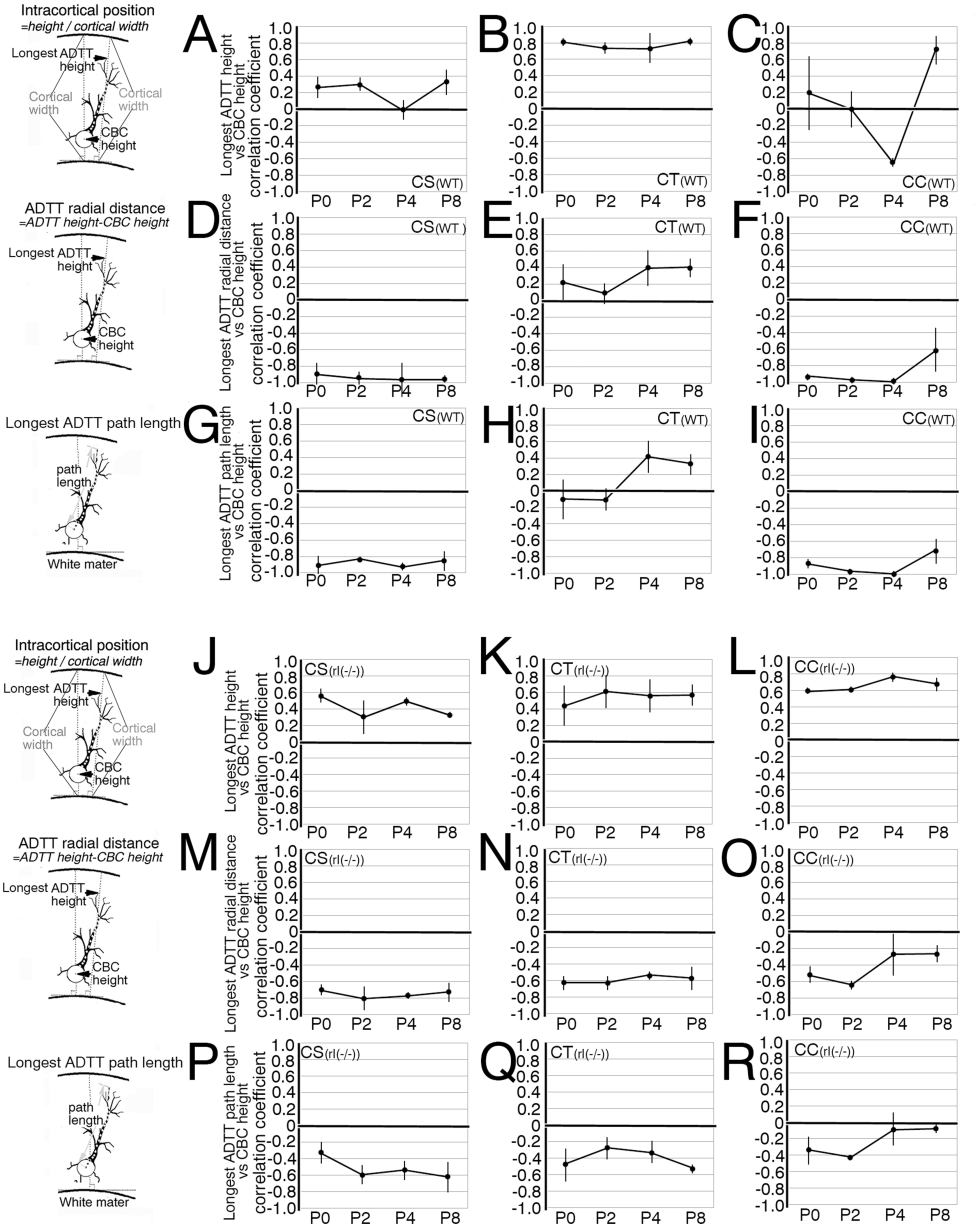
Developmental changes of the correlation coefficients in three measured types for each projection neuron form P0 until P8. **(A–C,J–L)** The average of the correlation coefficients between cell body center (CBC) height per cortical width and the longest apical dendrite terminal tip (ADTT) height with the longest path from CBC to ADTT height per cortical width in wild-type (WT) corticospinal neurons (CS) **(A)**, corticothalamic neurons (CT) **(B)**, and corticocallosal neurons (CC) **(C)**, and in reeler [rl(−/−)] CS **(J)**, CT **(K)**, and CC **(L)**. **(D–F,M–O)** Between CBC height per cortical width and the longest ADTT radial distance in wild-type CS **(D)**, CT **(E)**, and CC **(F)**, and reeler CS **(M)**, CT **(N)**, and CC **(O)**. **(G–I,P–R)** Between CBC height per cortical width and the longest ADTT path length in wild-type CS **(G)**, CT **(H)**, and CC **(I)**, and reeler CS **(P)**, CT **(Q)**, and CC **(R)**. Error bars show 95% confidence intervals from three mice. Schematic illustration of the measured parameters drawn on the left sides of **A,D,G,J,M,P**.

Developmental changes in the correlations between intracortical longest ADTT positions, longest ADTT radial distances, and longest ADTT path lengths with intracortical CBC positions were evaluated in wild-type CS, CT, and CC neurons (Figures 8A–I; Supplementary Figures 9A–C, 10A–C, 11A–C). From P0 to P8, the ADTTs of wild-type CS neurons reached beneath the pia matter, regardless of CBC position (Figures 8A; Supplementary Figure 9A). This significant correlation between longest ADTT radial distances and CBC positions showed that CS neurons located at deeper positions had larger longest ADTT radial distances, mirroring the ADTT locations of CS neurons (Figures 8D,G; Supplementary Figures 9A, 10A). These features were similarly observed in wild-type CC neurons from P0 to P4; however, much of the CC neurons with CBCs located at 0.6 (CBC height / cortical width) retained their longest ADTTs at approximately 0.8 at P8 (Figures 8F,I; Supplementary Figure 9C). These findings were reflected in the radial distances of longest ADTT of CC neurons, wherein those positioned at 0.6 at P8 demonstrated shorter measurements than those at P4 and showed a positive correlation with CBC position (Supplementary Figure 10C). For wild-type CT neurons, those with CBCs positioned deeper than 0.2 showed ADTTs terminating in the middle zone of M1 from P0 to P2 (arrowhead in Figure 6B), whereas the other labeled CT neurons extended their longest ADTTs beneath the pia matter. It should be noted that the DiI-labeled neurons comprised CT and subplate neurons from P0 to P8 (McConnell et al., 1989). The deeper-positioned, mid-cortical terminating population likely represented subplate neurons, a large portion of which showed ADTT within the cortex (Hoerder-Suabedissen and Molnár, 2012). From P4 to P8, CT neurons with CBCs positioned at 0.2 had ADTTs terminating at approximately 0.7, and those at 0.5 had ADTTs that extended beneath the pia matter (Supplementary Figure 9B).

Likewise, developmental changes in the same correlations were evaluated among reeler CS, CT, and CC neurons with CBCs that were scattered throughout the M1 (Figures 8J–R; Supplementary Figures 9D–F, 10D–F, 11D–F). From P0 to P2, all three types showed a significant correlation between longest ADTT radial distances and CBC positions (Figures 8M–O; Supplementary Figures 10D–F). Although deeper-positioned neurons extended their longest ADTTs with longer radial distances, some neurons in the upper half of the cortex oriented their longest ADTTs toward the white matter (Supplementary Figures 10D–F). However, unlike their wild-type counterparts, longest ADTTs did not accumulate in the opposite direction. Additionally, longest ADTT positions were largely dependent on CBC positions during this period (Figures 8J–L; Supplementary Figures 9D–F). Thereafter, reeler CS neurons displayed a strengthened correlation between longest ADTT radial distances and CBC positions (Figure 8M; Supplementary Figure 10D). These data indicated a tendency to congregate in a relatively narrow region within 0.6–1.0 (CBC height / cortical width). Conversely, reeler CC neurons showed no correlation between longest ADTT radial distances and CBC positions (Figure 8O; Supplementary Figure 10F). These data indicated dispersed longest ADTTs within a 400-μm radius from the CBC, with free radial distance and free path lengths regardless of CBC position (Figures 8O,R; Supplementary Figure 10F). Notably, from P4 to P8, the longest ADTT positions of reeler CC neurons became significantly correlated with CBC positions (Figure 8L; Supplementary Figure 9F). Finally, reeler CT neurons at P8 showed correlations of longest ADTT positions and longest ADTT radial distances with CBC positions (Figures 8K,N; Supplementary Figures 9E, 10E). This implies that reeler CT neurons were likely a mixed population containing neurons exhibiting congregational and dispersive AD extension modes.

**Figure 9:**
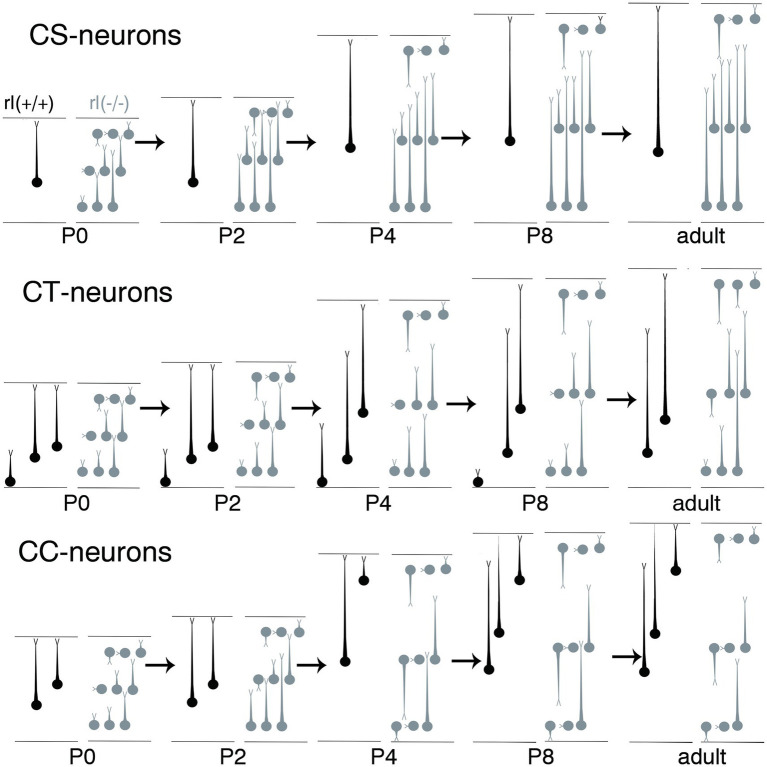
Schematic illustration of the developmental extensions of apical dendrites from corticospinal neurons (CS), corticothalamic neurons (CT), and corticocallosal neurons (CC) in the cerebral cortices of wild-type and reeler mice from P0 to P8. The area between the upper and lower horizontal lines represents the primary motor area of the cerebral cortex. The upper lines indicate the pia matter, and the lower lines indicate the border to the white matter. The circles indicate the cell bodies, and the processes extending from the cell bodies with short, thin terminal branches indicate the apical dendrites. The neurons drawn in black represent wild-type neurons, whereas those drawn in gray represent reeler neurons.

## 4 Discussion

### 4.1 Methodological considerations

In this study, the retrograde biocytin-labeling method or postmortem DiI technique were used to visualize CS, CT, and CC neurons. However, these methods have certain considerations that must be acknowledged. For CT neurons, biocytin and DiI injected into the VA + VL nucleus spread to the surrounding thalamic nuclei, potentially labeling layer 5 neurons projecting to non-specific nuclei and layer 6 neurons projecting to the other nucleus than VA + VL nucleus. Moreover, the postmortem DiI technique labeled subplate neurons projecting to the thalamus from P0 to P8. For CC neurons, biocytin and DiI, injected into the all layers of left M1 region, labeled those in layer 2 + 3 and layer 5 and 6. These considerations necessitated the analysis of labeled neurons from each injection site as a single group.

### 4.2 Two patterns in ADTT extension of CS, CT, and CC neurons within the cerebral cortices of reeler mutant mice

For CS neurons, the adult cerebral cortices of reeler mutant mice revealed congregation patterns in AD arborization, wherein their ADTTs congregated in a specific region in the upper cortical zone regardless of CBC position. In contrast, wild-type cortices presented with ADTTs reaching the narrow outer cortical zone. For CC neurons, reeler brains demonstrated a dispersion pattern in AD arborization, wherein the ADTT positions were dispersed at a 300-μm radius from the CBC position. Their wild-type counterparts, on the contrary, were positioned in layer 2 + 3 with ADTTs reaching the narrow outer zone, and in layer 5 and 6 with ADTTs within layer 1, 2 + 3, 4 or layer 5 (Koester and O’Leary, 1992; Harris and Shepherd, 2015). For CT neurons, reeler brains exhibited a hybrid presentation of the previous two neuronal patterns. However, wild-type brains comprised neurons positioned in layer 5 and 6, where the former extended their ADTTs until beneath the pia matter, whereas the latter terminated their ADTTs at layer 2 + 3, 4 and 5. As wild-type CT neurons in layer 5 are thought to be the driver neurons to high-order thalamic nuclei with thick tufted ADs (Sherman and Guillery, 2003), the congregation-type reeler CT neurons may correspond to the wild-type CT neurons in layer 5. Accordingly, the dispersion-type reeler CT neurons may correspond to the wild-type CT neurons in layer 6. Further studies analyzing reeler CT neurons based on layer-specific molecular markers are warranted for more precise comparisons (Katsuyama and Terashima, 2009; Boyle et al., 2011).

In the cerebral cortex of wild-type mice, projection neurons receive inputs through projection-target specific synaptic organization, which regulates the extension pattern in AD arborization (White and Hersch, 1982; Porter and White, 1986; Harris and Shepherd, 2015; Kawaguchi, 2017). CS neurons have thick-tufted ADs, whereas layer 5 CC neurons have slender ADs (Connors and Gutnick, 1990; Reiner et al., 2003; Harris and Shepherd, 2015; Kawaguchi, 2017). The extension pattern of AD arborization in projection neurons is associated with intrinsic electrophysiological properties; CS neurons and layer 5 CT neurons exhibit a bursting firing pattern, whereas CC neurons and layer 6 CT neurons show a regular spiking pattern (Connors and Gutnick, 1990; Larkum et al., 2003, 2007; Llano and Sherman, 2009; Ramaswamy and Markram, 2015; Stuart and Spruston, 2015; Kawaguchi, 2017). In the cerebral cortex of reeler mutant mice, CS neurons and some CT neurons extend AD in a congregation pattern, while CC neurons and the remaining CT neurons extend in a dispersion pattern. Furthermore, the spatially restricted accumulation of three distinct types of ADTTs derived from single neuron of reeler CS, CT, and CC neurons suggests that ADs are capable of forming efficient synaptic contacts with presynaptic terminals localized within a defined region. Although reeler cortices exhibit malposition of projection neurons and abnormal pathways of thalamocortical fibers (Caviness and Frost, 1983; Terashima et al., 1983; Terashima et al., 1985), the global and local neural circuits in reeler cortices are largely unchanged compared to wild-type cortices (Guy and Staiger, 2017). The projection target-specific pattern of AD extension is suggested to reflect the formation of projection target-specific synaptic connections with afferent fiber inputs.

### 4.3 Development of AD arborization in CS, CC, and CT neurons

In wild-type brains at P0, the ADs of CS, CT, and CC neurons, which were directly transformed from the leading processes during the terminal phase of neuronal migration, were attached to the outer cortical layers (Olson et al., 2006; O’Dell et al., 2015). Conversely, in reeler brains, neuroblasts tended to stop migrating at the borders of the internal plexiform zone, which is mainly composed of precursor dendrites. This disrupts their contact with Cajal–Retzius cells, hindering AD extension to the pia mater. Consequently, these neurons initiate AD development toward the internal plexiform zone, with those positioned above the zone exhibiting a downward orientation and those positioned below exhibiting an upward orientation on embryonic days 15–16 (IPZ at E15-E16) (Pinto Lord and Caviness, 1979; Tabata and Nakajima, 2002; O’Dell et al., 2015). Furthermore, ADTTs within the cortices of reeler mutant mice accumulate densely in the internal plexiform zone and progressively move beyond thereafter (Pinto Lord and Caviness, 1979). These patterns were commonly found in all three neuron types of reeler brains at P0. Additionally, although the radial distances of their ADTTs correlated with CBC positions, ADTT positions also exhibited a correlation with CBCs. Compared to wild-type cortices, the distribution of reeler neuron ADTTs was significantly more widespread, which is likely due to the disruptions in the gradient of semaphorin 3A (Sema3A), a chemoattractant for cortical AD (Polleux et al., 2000; Katsuyama and Terashima, 2009).

From P0 to P8, reeler CS and CC neurons demonstrated distinct AD extension patterns, forming congregation and dispersion configurations, respectively (Figure 9). During the first two postnatal weeks, projection neurons in the cerebral cortex establish target-specific synaptic connections through the formation and subsequent pruning of surplus connections on their soma–dendritic compartments (Yoshimura et al., 2005; Kampa et al., 2006; Le Bé and Markram, 2006; Thomson and Lamy, 2007; Brown and Hestrin, 2009; Faust et al., 2021). Accordingly, AD arborization becomes influenced in a target-specific manner (White and Hersch, 1982; Porter and White, 1986; Larkman, 1991a,b,c; Larkman et al., 1992; Kasper et al., 1994a,b,c). In wild-type cortices, CS neurons, CC neurons in layer 2 + 3 and upper layer 5, and CT neurons in layer 5 extend their ADTTs to the outer zone from P0 to P8 by elongating terminal tips from the distal dendrite regions. However, CC neurons in deep layer 5 and 6, and CT neurons in layer 6 retract their distal AD, resulting in their localization in layer 2 + 3 or layer 4 (Koester and O’Leary, 1992; Kumar and Ohana, 2008; Llano and Sherman, 2009). Considering that the global and local neural circuitry within the reeler cortex is largely common compared to wild-type cortex (Guy and Staiger, 2017), it is likely that from P0 to P8 the AD extension of reeler projection neurons is influenced by a common mechanism with wild-type counterparts.

### 4.4 Limitations

Wild-type CC and CT neurons are sub-grouped into locating layer 2 + 3 and layer 5 neurons, and locating layer 5 and layer 6 neurons, respectively. Different subgroups seem to exert distinctive features. In this study, where the neurons are identified by retrograded labeling alone, the labeled reeler CT and CC neurons could not be classified into subgroups. Hence, it is inconclusive whether the CC neurons in different layer are all dispersion-type, whether CT neurons in layer 5 are congregation-type, or whether CT neurons in layer 6 are dispersion-type. Currently, the molecules specifically expressed by neurons in different layers have been identified, which, in combination with retrograde labeling, could be used in the future to provide more discriminating data.

## 5 Conclusion

In the cerebral cortices of reeler mutant mice, CS neurons exhibited a congregation pattern in ADTT extension, with a dense accumulation in a specific upper cortical zone. CC neurons exhibited a dispersion pattern in ADTT extension, where the tips were scattered within a 300-μm radius from their CBCs. CT neurons displayed a hybrid pattern of congregation- and dispersion-type neurons. Although the apical dendrites of these three neuron types originate from the same projection neurons in reeler cortices, the study showed that their arborization can follow a congregation or dispersion pattern depending on the formation of the projection target-specific neuronal connections from P0 to P8. This study revealed the mechanism in the developmental generation of the characteristic dendritic shape exerting distinctive function, which is veiled in wild-type mice. In the future, the molecular mechanisms of dispersion- and congregation-type extension of ADs should be studied.

## Data Availability

The original contributions presented in the study are included in the article/Supplementary material, further inquiries can be directed to the corresponding author.
