# Supplementary material for: Two patterns in apical dendrite extensions of projection neurons within cerebral cortex of reeler mutant mice
Source: Front Neuroanat. 2025 May 30;19:1560972. doi: 10.3389/fnana.2025.1560972 (PMC12162585; doi:10.3389/fnana.2025.1560972)
Supplement: Supplementary file 1 [file Data_Sheet_1.pdf]

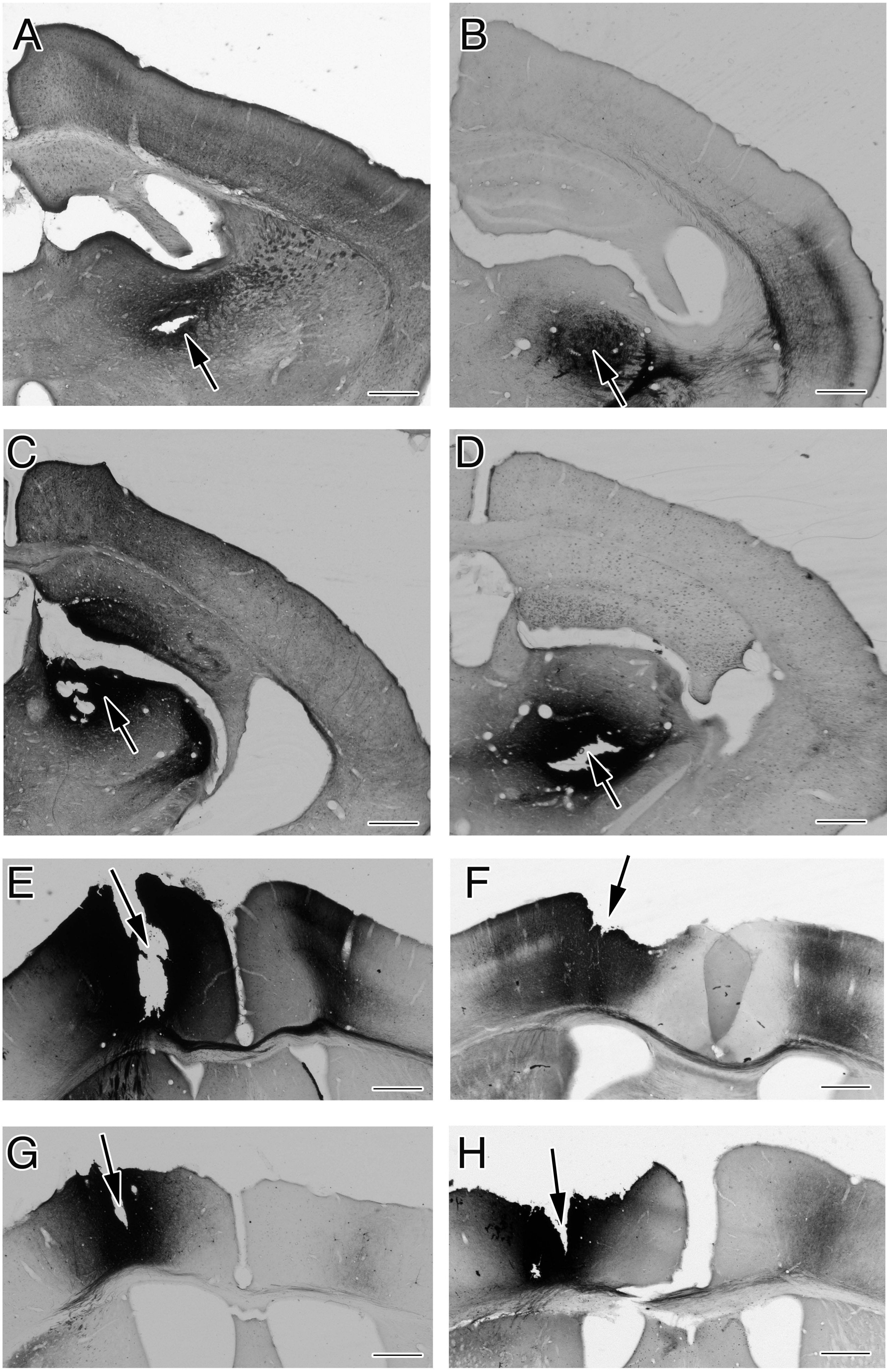
A
B
C
D
E
F
G
H

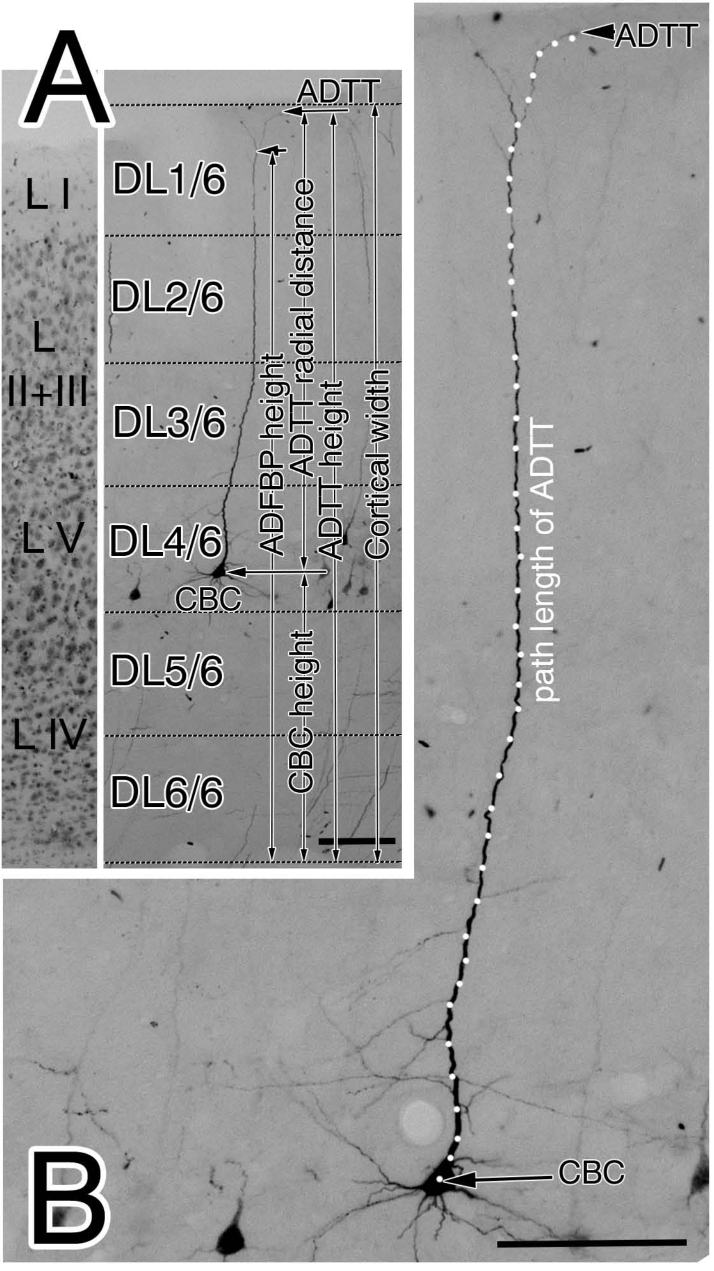

A
L I
L
II+III
L V
L IV
DL1/6
DL2/6
DL3/6
DL4/6
DL5/6
DL6/6
ADTT
ADFBP height
ADTT radial distance
ADTT height
Cortical width
CBC
CBC height
ADTT
path length of ADTT
CBC
B

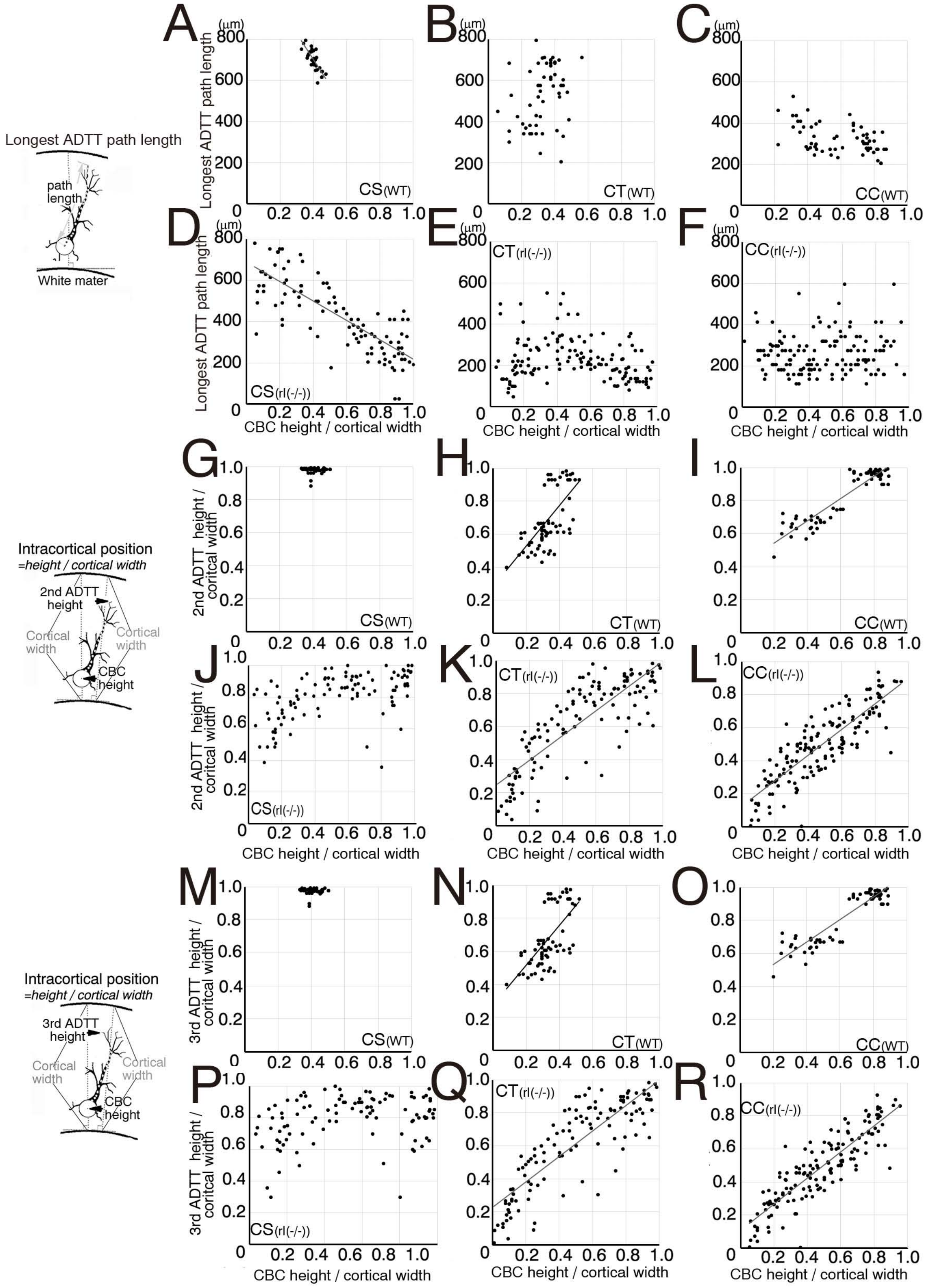

Longest ADTT path length
path length
White mater
Intracortical position
=height / cortical width
2nd ADTT height
Cortical width
Cortical width
CBC height
Intracortical position
=height / cortical width
3rd ADTT height
Cortical width
Cortical width
CBC height
A
B
C
D
E
F
G
H
I
J
K
L
M
N
O
P
Q
R
(µm)
Longest ADTT path length
2nd ADTT height / coritcal width
3rd ADTT height / coritcal width
CBC height / cortical width
CS(WT)
CT(WT)
CC(WT)
CS(rl(-/-))
CT(rl(-/-))
CC(rl(-/-))
0
200
400
600
800
0.2
0.4
0.6
0.8
1.0

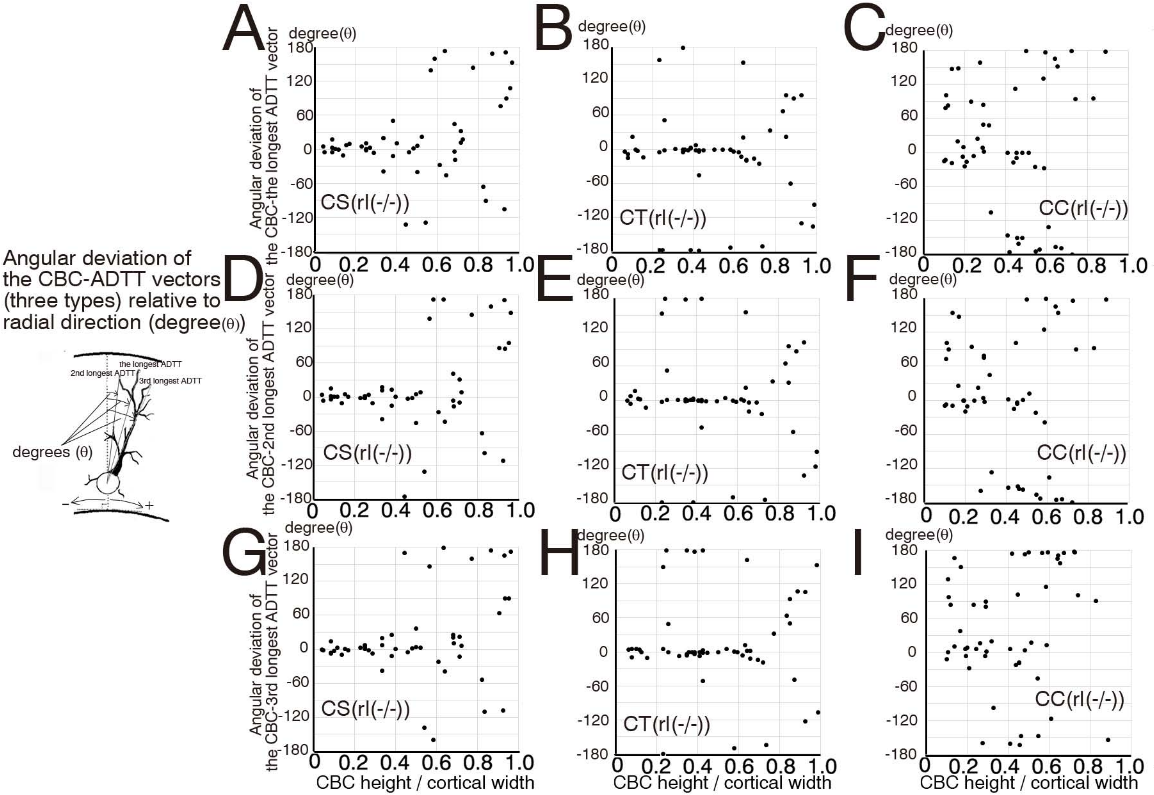

A
B
C
D
E
F
G
H
I
Angular deviation of the CBC-ADTT vectors (three types) relative to radial direction (degree(θ))
the longest ADTT
2nd longest ADTT
3rd longest ADTT
degrees (θ)
Angular deviation of the CBC-the longest ADTT vector
Angular deviation of the CBC-2nd longest ADTT vector
Angular deviation of the CBC-3rd longest ADTT vector
degree(θ)
180
120
60
0
-60
-120
-180
0
0.2
0.4
0.6
0.8
1.0
CS(rl(-/-))
CT(rl(-/-))
CC(rl(-/-))
CBC height / cortical width

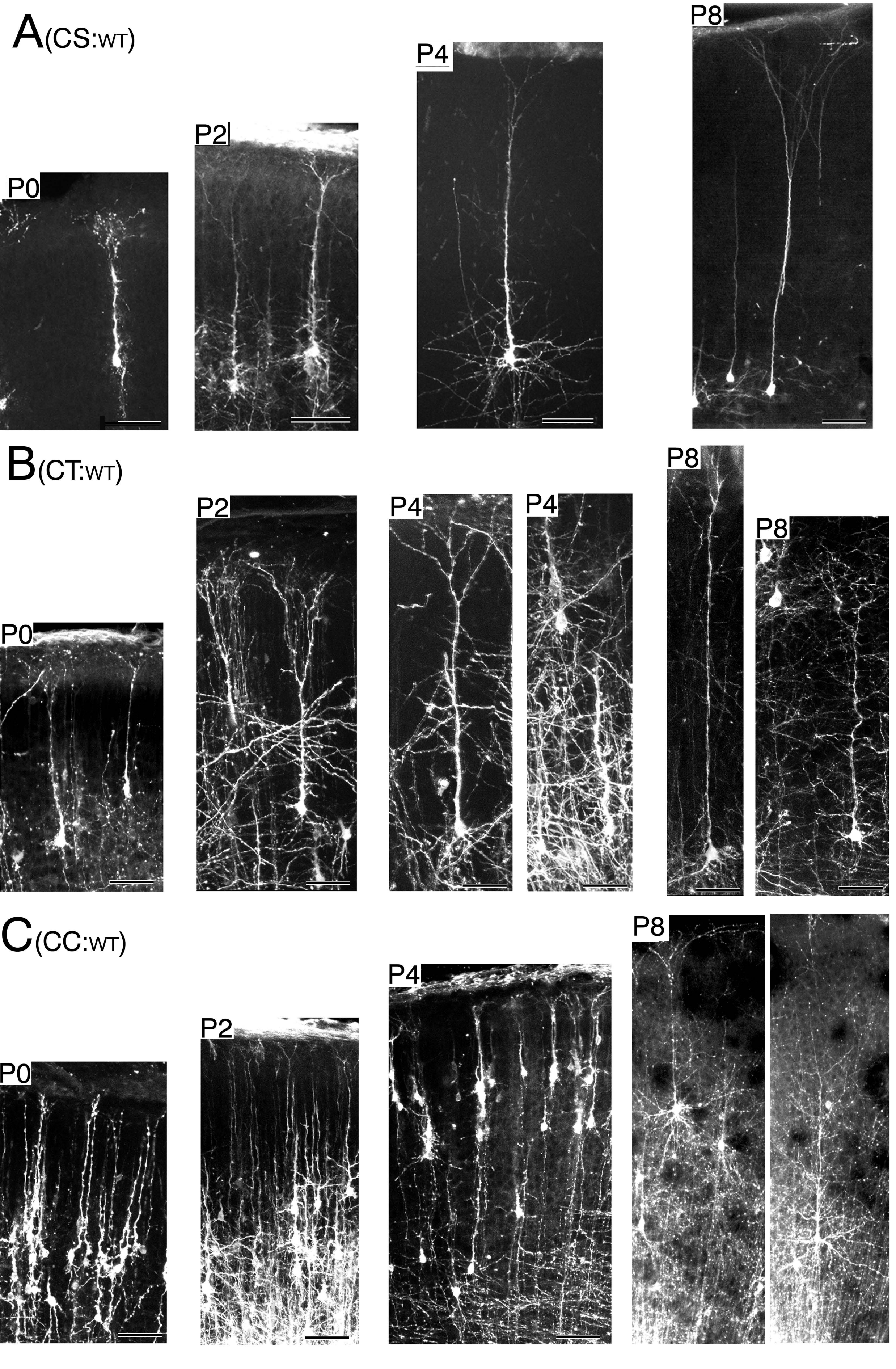
A(CS:WT)
P0
P2
P4
P8
B(CT:WT)
P0
P2
P4
P4
P8
P8
C(CC:WT)
P0
P2
P4
P8

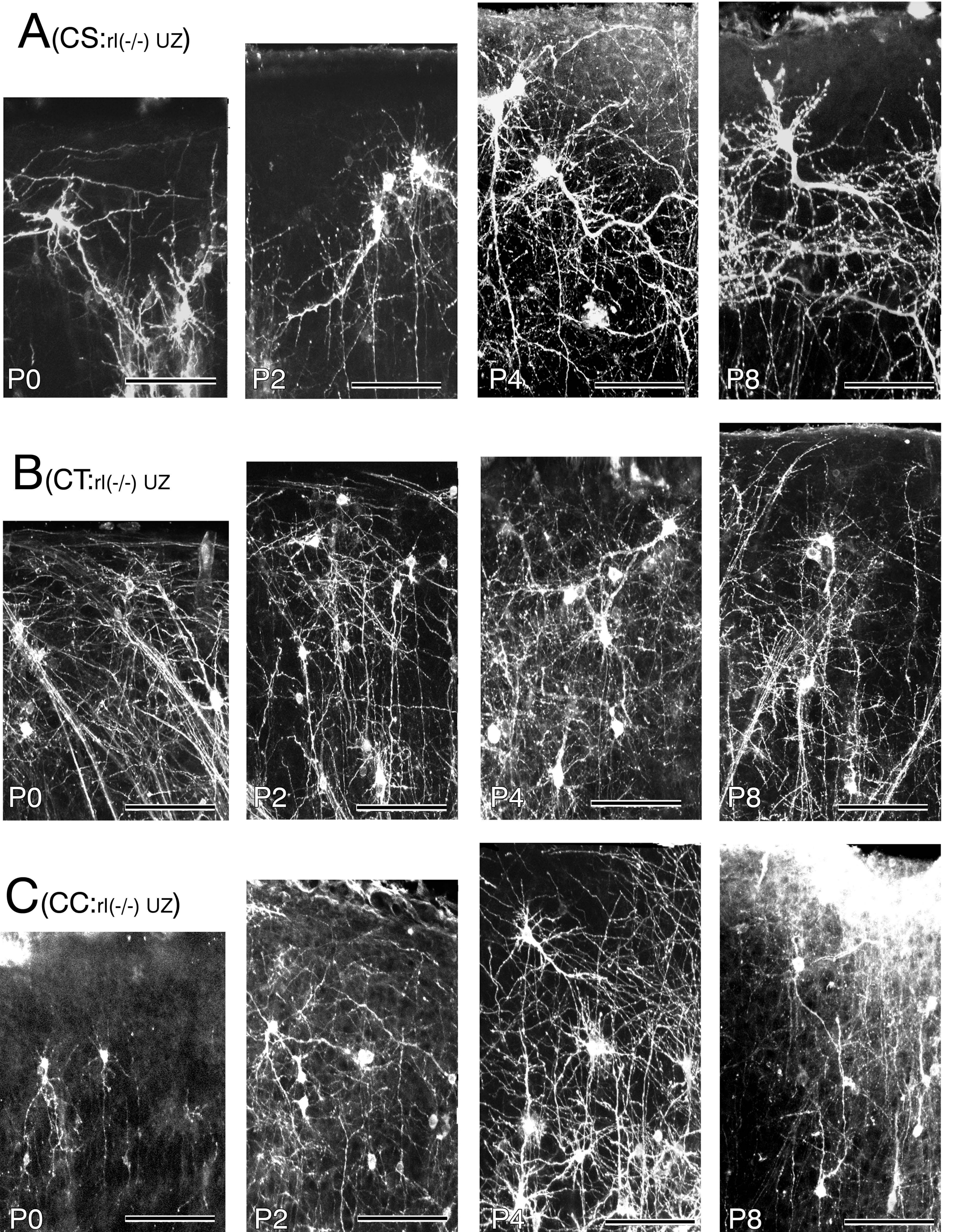
A(CS:rl(-/-) UZ)
P0
P2
P4
P8
B(CT:rl(-/-) UZ
P0
P2
P4
P8
C(CC:rl(-/-) UZ)
P0
P2
P4
P8

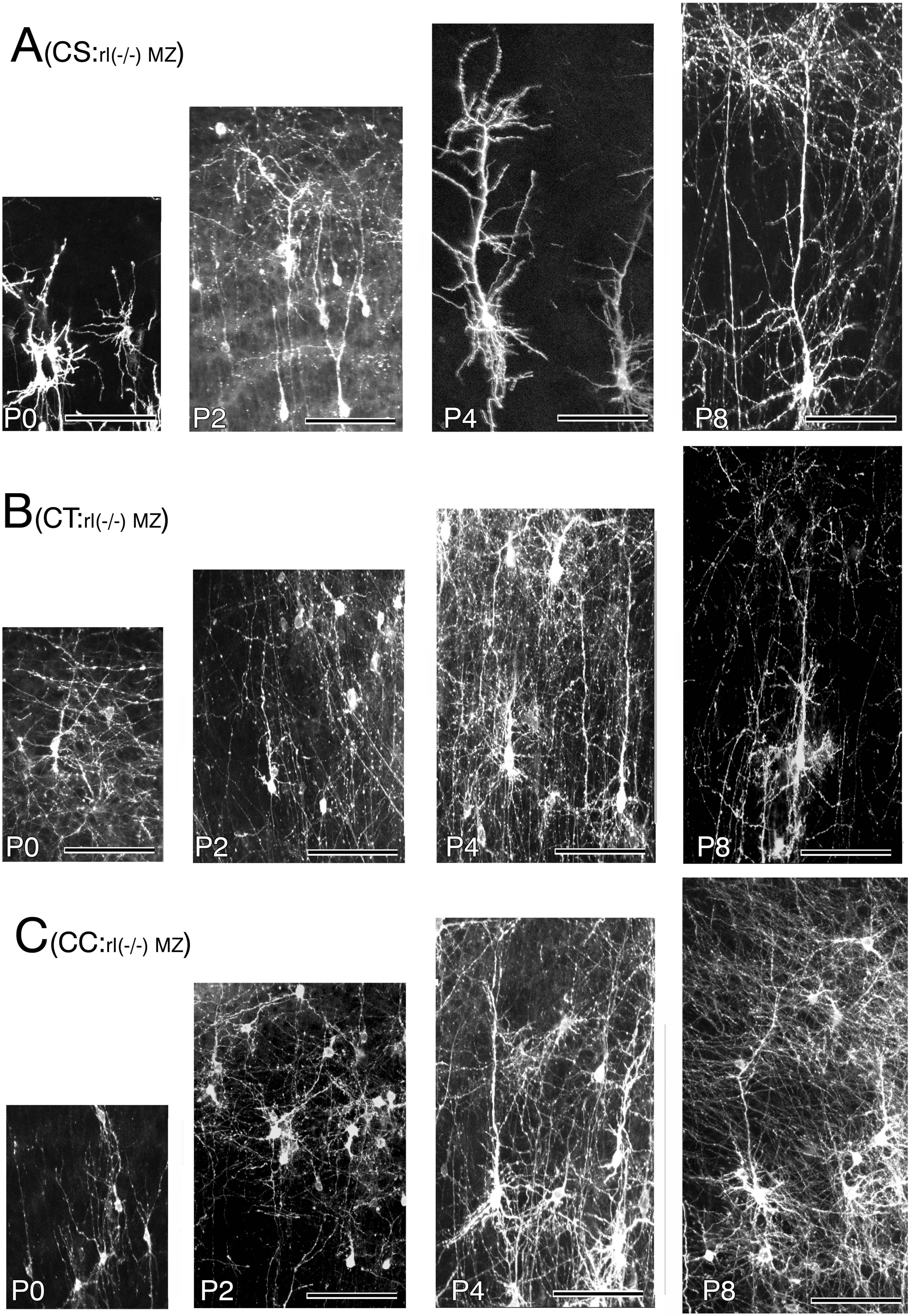
A(CS:rl(-/-) MZ)
P0
P2
P4
P8
B(CT:rl(-/-) MZ)
P0
P2
P4
P8
C(CC:rl(-/-) MZ)
P0
P2
P4
P8

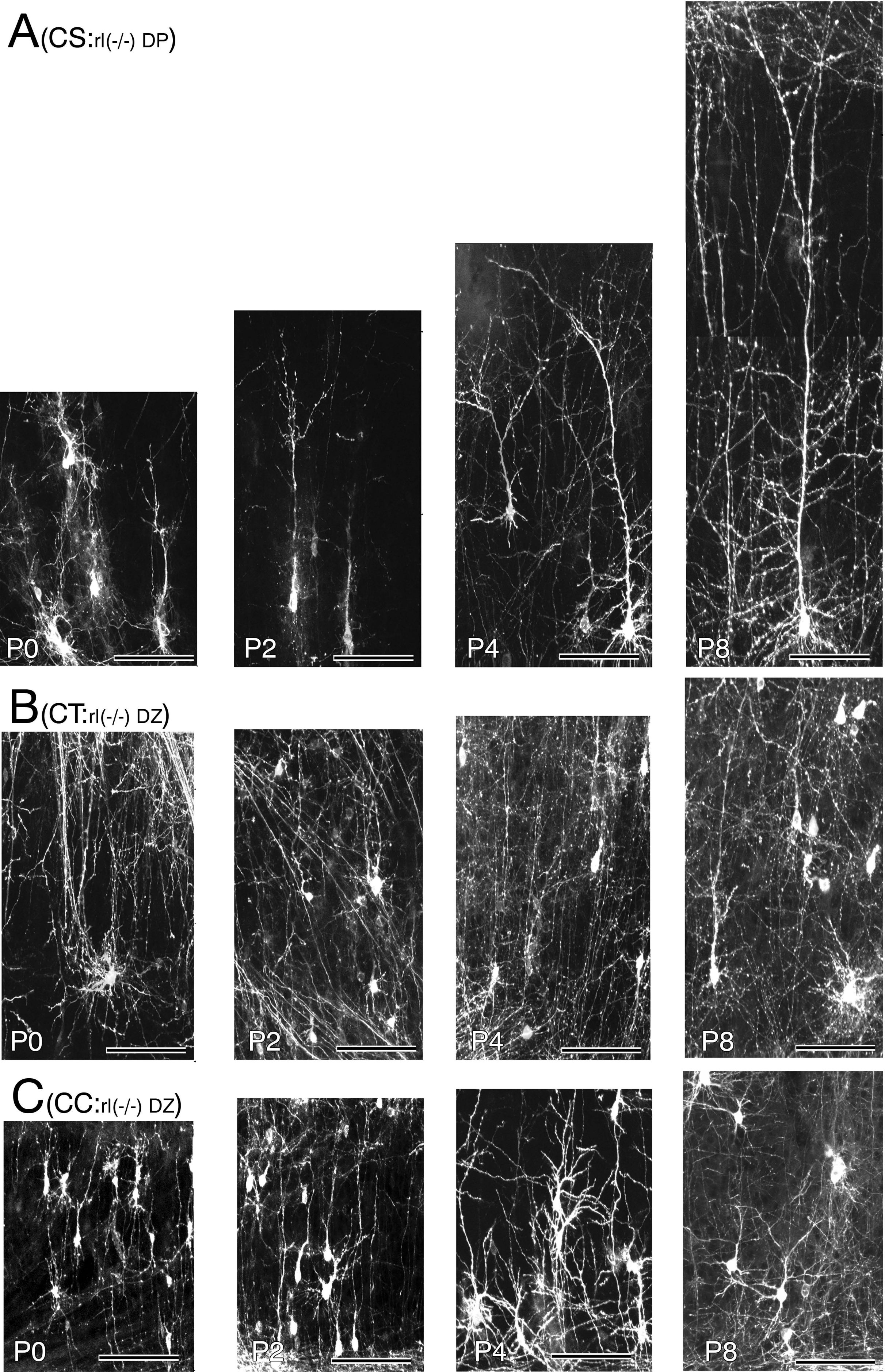
A(CS:rl(-/-) DP)
P0
P2
P4
P8
B(CT:rl(-/-) DZ)
P0
P2
P4
P8
C(CC:rl(-/-) DZ)
P0
P2
P4
P8

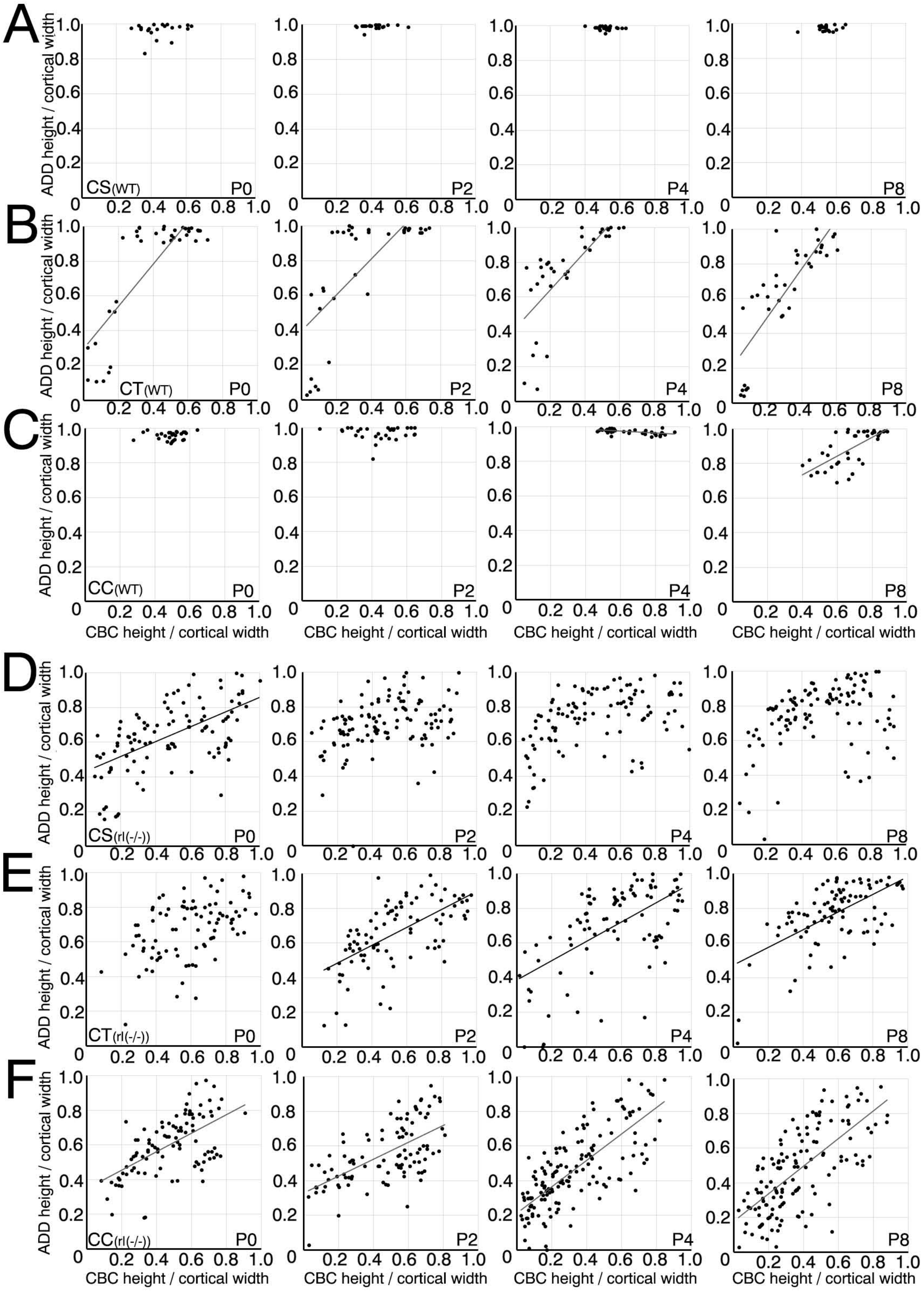
A
B
C
D
E
F
ADD height / cortical width
CBC height / cortical width
CS(WT)
CT(WT)
CC(WT)
CS(rl(-/-))
CT(rl(-/-))
CC(rl(-/-))
P0
P2
P4
P8
0
0.2
0.4
0.6
0.8
1.0

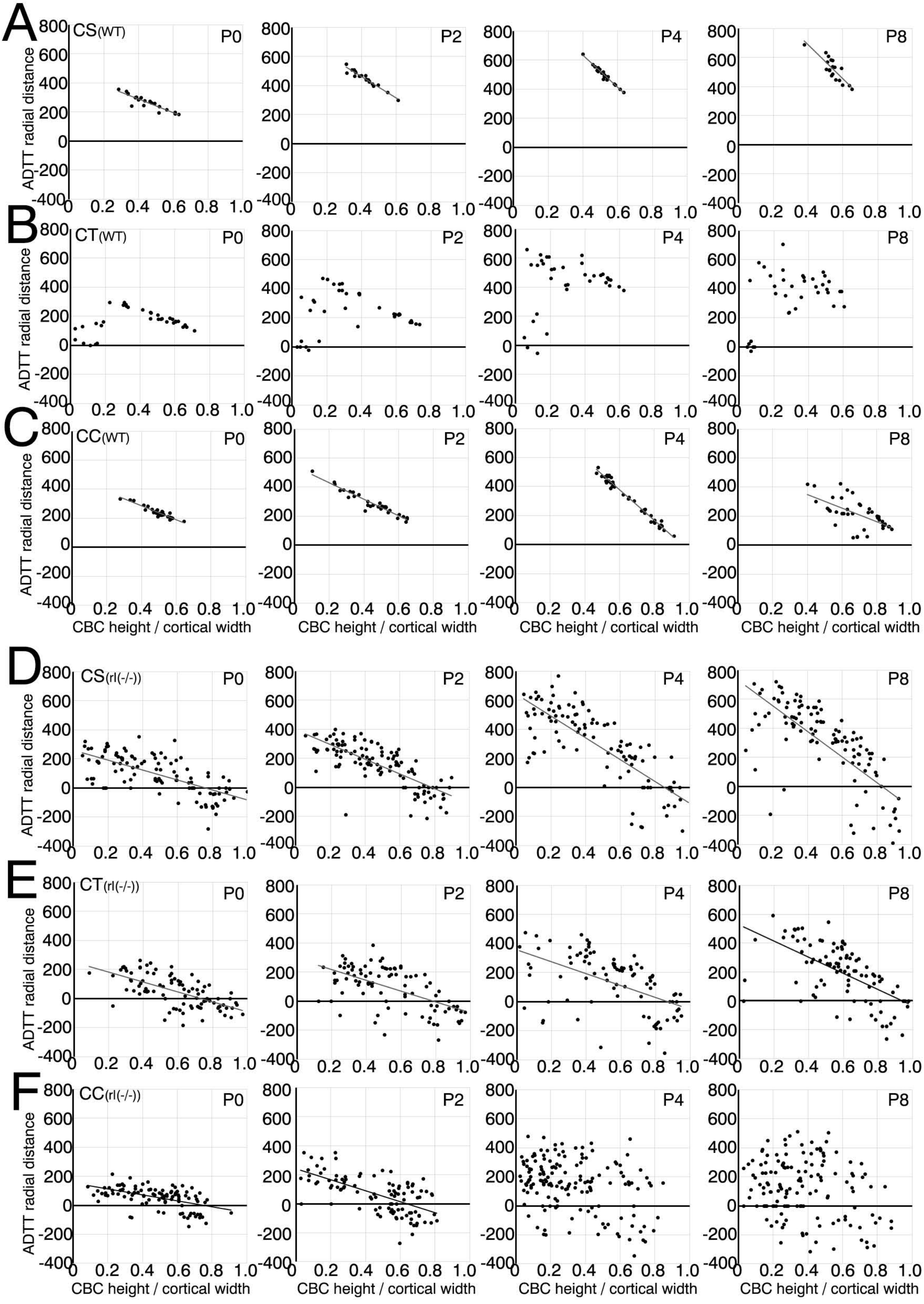

A
B
C
D
E
F
CS(WT)
CT(WT)
CC(WT)
CS(rl(-/-))
CT(rl(-/-))
CC(rl(-/-))
P0
P2
P4
P8
ADTT radial distance
CBC height / cortical width

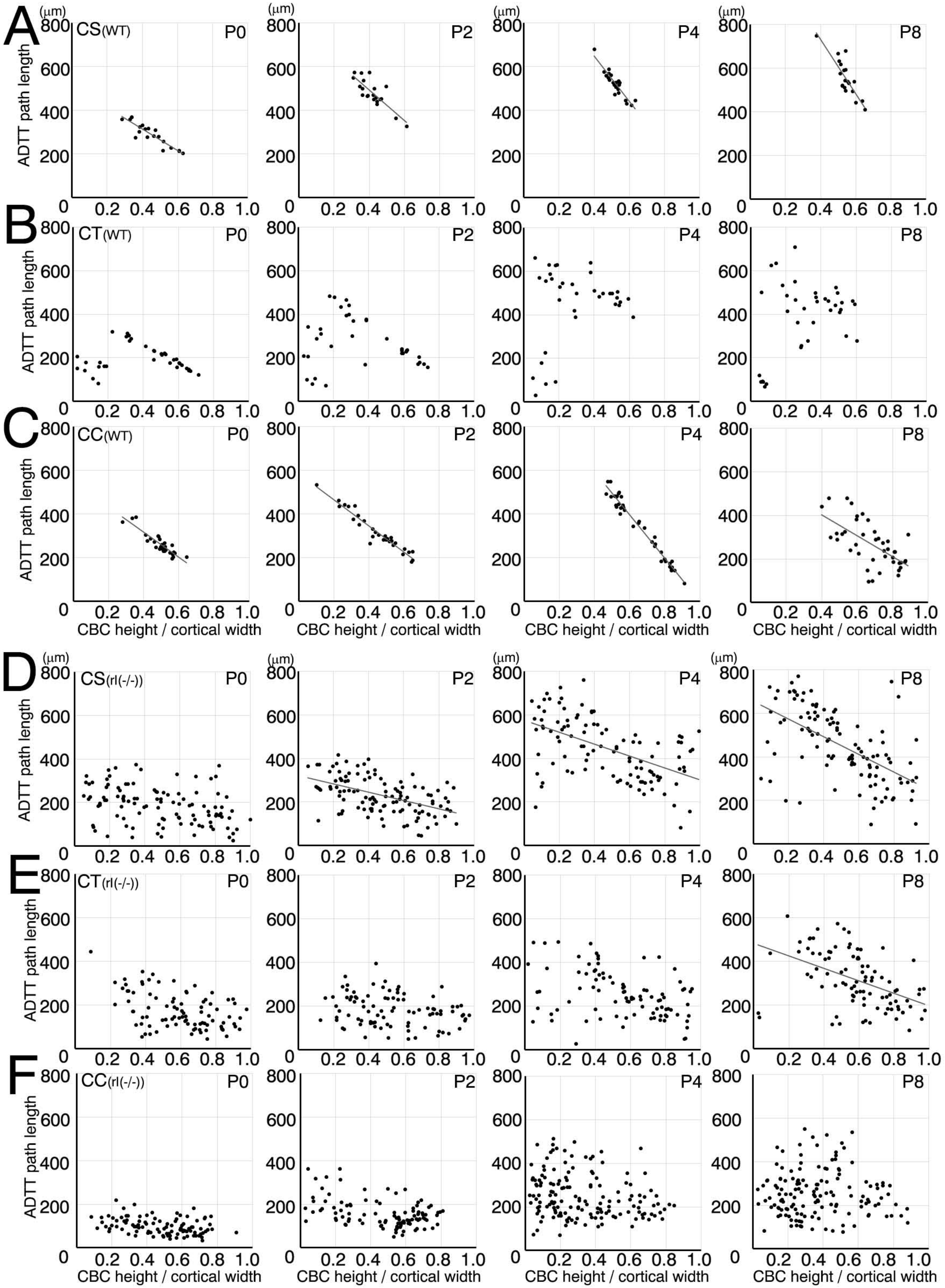
A
B
C
D
E
F
CS(WT)
CT(WT)
CC(WT)
CS(rl(-/-))
CT(rl(-/-))
CC(rl(-/-))
P0
P2
P4
P8
(µm)
ADTT path length
CBC height / cortical width
0
200
400
600
800
0.2
0.4
0.6
0.8
1.0

Supplementary Figure legends

Supplementary Figure 1. Injection sites of biocytin in the remaining two wild-type mice (WT) (A–B, E-F) and two reeler mutant mice (rl(-/-)) (C-D, G-H). (A-D) Coronal secstions including the ventral anterior and ventral lateral (VA+VL) nucleus of the right thalamus to label corticothalamic neurons (CT). (E-H) Coronal sections including the left primary motor cortex (M1) label corticocallosal neurons (CC). Arrows indicate injection sites. Note. Biocytin is injected to all layers of left M1. Scale bar: 500 μm.

Supplementary Figure 2. Divided six layers and measured parameters in wild-type the right primary motor cortex (M1) with a corticospinal neuron. (A) Divided six layers M1 (from the pia matar, DL1/6, DL2/6, DL3/6, DL4/6, DL5/6, DL6/6). Appended with counterstained adjacent field of the same section. Note. DL1/6 is largely corresponded to layer 1. DL2/6 and upper part of DL3/6 is to layer 2+3. Deeper part of DL3/6, DL4/6 and upper part of DL5/6 is to layer 5. Deeper part of DL5/6 and DL6/6 is to layer 6. The height of cell body center (CBC height), the height of longest apical dendrite terminal tip (ADTT) with the longest path length from CBC to ADTT, the height of final branching point of main shaft of AD (ADFBP), the radial distance of longest ADTT, the width of M1 (cortical width) are drawn in the six-divided M1. (B) Enlarged image of the corticospinal neuron in (A). White dot lines indicate the path length of ADTT from CBC. Scale bar: 100 μm.

Supplementary Figure 3. Scatter diagrams of three measured types for each projection neuron. (A-F) Between cell body center (CBC) height per cortical width and the longest ADTT path length in wild-type (WT) corticospinal neurons (CS) (A), corticothalamic neurons (CT) (B), and corticocallosal neurons (CC) (C), and in reeler (rl(-/-)) CS (D), CT (E), CC (F). (G-L) Between CBC height per cortical width and the second longest ADTT height per cortical width in WT CS (G), CT (H), and CC (I), and in rl(-/-) CS (J), CT (K), and CC (L). (M-R) Between CBC height per cortical width and the third longest ADTT height per cortical width in WT CS (M), CT (N), and CC (O), and in rl(-/-) CS (P), CT (Q), and CC (R). When the correlation coefficient exceeded 0.5 in three mice, regression lines were drawn using this formula. Schematic illustration of the measured parameters drawn on the left sides of between A and D, and G and H.

Supplementary Figure 4. Scatter diagrams of three measured types for each projection neuron. (A-C) Between cell body center (CBC) height per cortical width and angular deviation of the CBC-the longest ADTT vector to relative to radial direction in reeler (rl(-/-)) corticospinal neurons (CS) (A), corticothalamic neurons (CT) (B), and corticocallosal neurons (CC) (C). (D-F) Between cell body center (CBC) height per cortical width and angular deviation of the CBC-the second longest ADTT vector to relative to radial direction in rl(-/-) CS (D), CT (E), and CC (F). (G-I) Between cell body center (CBC) height per cortical width and angular deviation of the CBC-the third longest ADTT vector to relative to radial direction in rl(-/-) CS (G), CT (H), and CC (I). Schematic illustration of the measured parameters drawn on the left sides

Supplementary Figure 5. Enlarged postmortem DiI-retrograde-labeled neurons in the right primary motor cortex of wild-type mice (WT) from P0 to P8. (A) Corticospinal neurons (CS). (B) Corticothalamic neurons (CT). (C) Corticocallosal neurons (CC). Scale bar: 100 μm.

Supplementary Figure 6. Enlarged postmortem DiI-retrograde-labeled neurons in the upper zone (UZ) of right primary motor cortex of reeler mice (rl(-/-)) from P0 to P8. (A) Corticospinal neurons (CS). (B) Corticothalamic neurons (CT). (C) Corticocallosal neurons (CC). Scale bar: 100 μm.

Supplementary Figure 7. Enlarged postmortem DiI-retrograde-labeled neurons in the middle zone (MZ) of right primary motor cortex of reeler mice (rl(-/-)) from P0 to P8. (A) Corticospinal neurons (CS). (B) Corticothalamic neurons (CT). (C) Corticocallosal neurons (CC). Scale bar: 100 μm.

Supplementary Figure 8. Enlarged postmortem DiI-retrograde-labeled neurons in the deep zone (DZ) of right primary motor cortex of reeler mice (rl(-/-)) from P0 to P8. (A) Corticospinal neurons (CS). (B) Corticothalamic neurons (CT). (C) Corticocallosal neurons (CC). Scale bar: 100 μm.

Supplementary Figure 9. Scatter diagrams from the three mice for each type of projection neurons between the cell body center (CBC) height per cortical width and lonest apical dendrite terminal tip (ADTT) height per cortical width in wild-type (WT) corticospinal neurons (CS) (A), corticothalamic neurons (CT) (B), and corticocallosal neurons (CC) (C), and in reeler (rl (-/-)) CS (D), CT (E), and CC (F) from P0 to P8. When the correlation coefficient exceeded 0.5 in three mice, regression lines were drawn using this formula.

Supplementary Figure 10. Scatter diagrams from the three mice for each type of projection neurons between the cell body center (CBC) height per cortical width and longest apical dendrite terminal tip (ADTT) radial distance in wild-type (WT) corticospinal neurons (CS) (A), corticothalamic neurons (CT) (B), and corticocallosal neurons (CC) (C), and in reeler (rl (-/-)) CS (D), CT (E), and CC (F) from P0 to P8. When the correlation coefficient exceeded 0.5 in three mice, regression lines were drawn using this formula.

Supplementary Figure 11. Scatter diagrams from the three mice for each type of projection neurons between the cell body center (CBC) height per cortical width and longest apical dendrite terminal tip (ADTT) path length in wild-type (WT) corticospinal neurons (CS) (A), corticothalamic neurons (CT) (B), and corticocallosal neurons (CC) (C), and in reeler (rl (-/-)) CS (D), CT (E), and CC (F) from P0 to P8. When the correlation coefficient exceeded 0.5 in three mice, regression lines were drawn using this formula.

Table 1. The number of neurons measured for three types of ADTTs and ADFBP in each of adult three mice.

	Corticospinal neurons	Corticothalamic neurons	Corticocallosal neurons
Wild-type	30, 32, 35	40, 43, 45	40, 44, 46
Reeler	44, 46, 53	43, 45, 47	40, 49, 68

Table 2. The number of neurons measured for longest ADTT in each of three mice from P0 to P8.

		Corticospinal neurons	Corticothalamic neurons	Corticocallosal neurons
P0	Wild-type	20, 20, 22	30, 32, 39	30, 30, 32
	Reeler	35, 35, 36	30, 31, 45	30, 35, 48
P2	Wild-type	25, 29, 30	30, 34 36	30, 32, 34
	Reeler	30, 40, 43	31, 37, 40	30, 36, 39
P4	Wild-type	32, 34, 37	33, 34, 34	35, 40, 41
	Reeler	32, 37, 44	30, 32, 40	36, 48, 46
P8	Wild-type	31, 35, 38	30, 36, 36	30, 33, 42
	Reeler	31, 32, 43	30, 32, 41	44, 45, 50
